# Platinum Nanoparticles on Metalloid Antimony Functionalized Graphitic Nanoplatelets for Enhanced Water Electrolysis

**DOI:** 10.1002/smll.202501408

**Published:** 2025-05-09

**Authors:** Do Hyung Kweon, Jae‐Hoon Baek, Sung O Park, Hyuk‐Jun Noh, Jong‐Pil Jeon, Jeong Hyeon Lee, Tae Joo Shin, Sang Kyu Kwak, In‐Yup Jeon, Jong‐Beom Baek

**Affiliations:** ^1^ School of Energy and Chemical Engineering/Center for Dimension‐Controllable Organic Frameworks Ulsan National Institute of Science and Technology (UNIST) Ulsan 44919 South Korea; ^2^ Hydrogen Fuel Cell Research Center Korea Institute of Science and Technology (KIST) Seoul 02792 Republic of Korea; ^3^ Department of Chemical and Biological Engineering Korea University Seongbuk‐gu Seoul 02841 Republic of Korea; ^4^ Graduate School of Semiconductor Materials and Devices Engineering Ulsan National Institute of Science and Technology (UNIST) UNIST‐gil 50 Ulsan 44919 Republic of Korea; ^5^ Department of Chemical Engineering Wonkwang University Iksandae‐ro 460 Iksan Jeonbuk 54538 Republic of Korea

**Keywords:** graphitic nanoplatelets, hydrogen evolution reaction, metalloid antimony functionalization, proton exchange membrane water electrolysis

## Abstract

Platinum (Pt) nanoparticles are considered to be the most efficient catalyst for acidic hydrogen evolution reaction (HER). However, they are expensive and unstable, because of agglomeration and Ostwald ripening. It is critically necessary for developing a better catalytic support to stabilize the Pt nanoparticles at low loading amounts. One efficient route to improving both catalytic activity and durability is metal catalysts stably anchored on heteroatom functionalized carbon supports via their strong interactions. Nevertheless, the interactions between “metallic” catalysts and “nonmetallic” heteroatom functionalized carbon supports are still unsatisfactory. Here, “metalloid” antimony (Sb) functionalized graphitic nanoplatelets (SbGnP) are reported to stably anchor Pt nanoparticles. The resulting Pt@SbGnP catalyst shows a record high acidic HER performance, attributable to the unique nature of Sb functional groups on SbGnP. Unlike typical low‐period nonmetallic heteroatoms on carbon supports, high‐period metalloid Sb with various oxidation states of SbO_x_ provided strong binding sites to stably anchor Pt nanoparticles, suppressing particle aggregation, and thus sustaining catalytic activity and stability.

## Graphical abstract

A new study enhances hydrogen production efficiency using platinum nanoparticles on antimony‐functionalized graphitic nanoplatelets. Functionalizing high‐period elements, a previously underexplored method, boosts catalytic activity and stability. This innovation paves the way for cost‐effective hydrogen production, crucial for clean energy, and offers valuable insights into designing better catalysts for sustainable energy solutions.

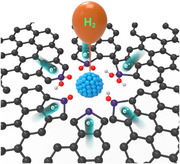

## 1 Introduction

Among renewable energy sources that could help achieve carbon neutrality, hydrogen (H_2_) energy receives continuing interest, because water is the only by‐product after reaction with oxygen.^[^ ^1^, ^2^, ^3^, ^4^ ^]^ Operating electrochemical water splitting technology with renewable energy sources provides a method of storing green energy in the form of H_2_ via the hydrogen evolution reaction (HER). The HER is an excellent route to produce high‐purity hydrogen from water electrolysis.^[^ ^5^, ^6^ ^]^ But to achieve practical performance, this process still requires substantial effort to discover breakthrough electrodes, which must satisfy efficiency, stability, and cost. An ideal catalyst must perform at minimum overpotential with large cathodic current density and high stability, to achieve enhanced HER performance.^[^ ^7^, ^8^ ^]^

To date, the most effective reported HER electrocatalysts are based on platinum (Pt) and its alloys, because of its rapid proton reduction kinetics and low overpotentials, for high energy conversion efficiency.^[^ ^9^ ^]^ However, because of its low availability and high cost, Pt is not suitable for commercial‐scale electrocatalysis.^[^ ^10^ ^]^ To maximize the use of Pt, extensive studies have been conducted to improve the catalyst performance by reducing the size of the Pt nanoparticles and thus maximizing the catalytic surface area on the support.^[^ ^11^ ^]^ Still, one of the major challenges is stabilizing Pt nanoparticles on the support for practical applications. It is because smaller particles, to minimize their surface energy, are prone to aggregating into larger particles by agglomeration and the Ostwald ripening process. This phenomenon results in serious catalytic activity decay.^[^ ^12^, ^13^ ^]^

To solve the problem, carbon‐based materials have been explored as efficient supports to stabilize Pt nanoparticles. The interaction between the catalytic nanostructures and the carbon supports also plays an important role in both the stability and catalytic performance, together with efficient electron transport.^[^ ^14^ ^]^ One promising way to achieve more efficient hydrogen generation, then, is to design anchoring sites on the carbon scaffolds, which can stabilize metal active sites. For this strategy, various advanced electrocatalysts have been developed by depositing Pt nanoparticles on functionalized CNT^[^ ^15^, ^16^ ^]^ and graphene.^[^ ^17^, ^18^ ^]^ Thus far, functionalizing nonmetallic heteroatoms, such as nitrogen (N) and phosphorous (P) in the group VA, has been reported to improve their interactions.^[^ ^18^, ^19^, ^20^ ^]^ Nevertheless, the performance of Pt nanoparticles on typically modified carbon supports remains unsatisfactory, because of the poor interaction between the “nonmetallic” elements on the supports and the “metallic” Pt nanoparticles.

In this work, we introduced antimony (Sb), which is one of the “metalloid” elements and belongs to the one of group VA elements, at the edge of graphitic nanoplatelets (SbGnP). Unlike widely used low‐period elements, such as nitrogen (N) and phosphorous (P), Sb is a high‐period element with partially occupied p‐ and empty d‐orbitals, which are expected to enhance the electron transfer ability between Pt nanoparticles and SbGnP support for an enhanced catalytic activity. It also forms various oxidation states of SbO_x_, which provide stronger binding sites for Pt nanoparticles for an improved stability.

As expected, the Pt nanoparticles on the SbGnP (Pt@SbGnP) catalyst showed outstanding electrocatalytic HER activity and stability. Theoretical calculations suggest that the intrinsic properties of the Sb functional groups provide strong interactions with Pt nanoparticles, inhibiting particle aggregation, while maintaining catalytic activity and thus stability.

## 2 Results and Discussion

**Figure 1:**
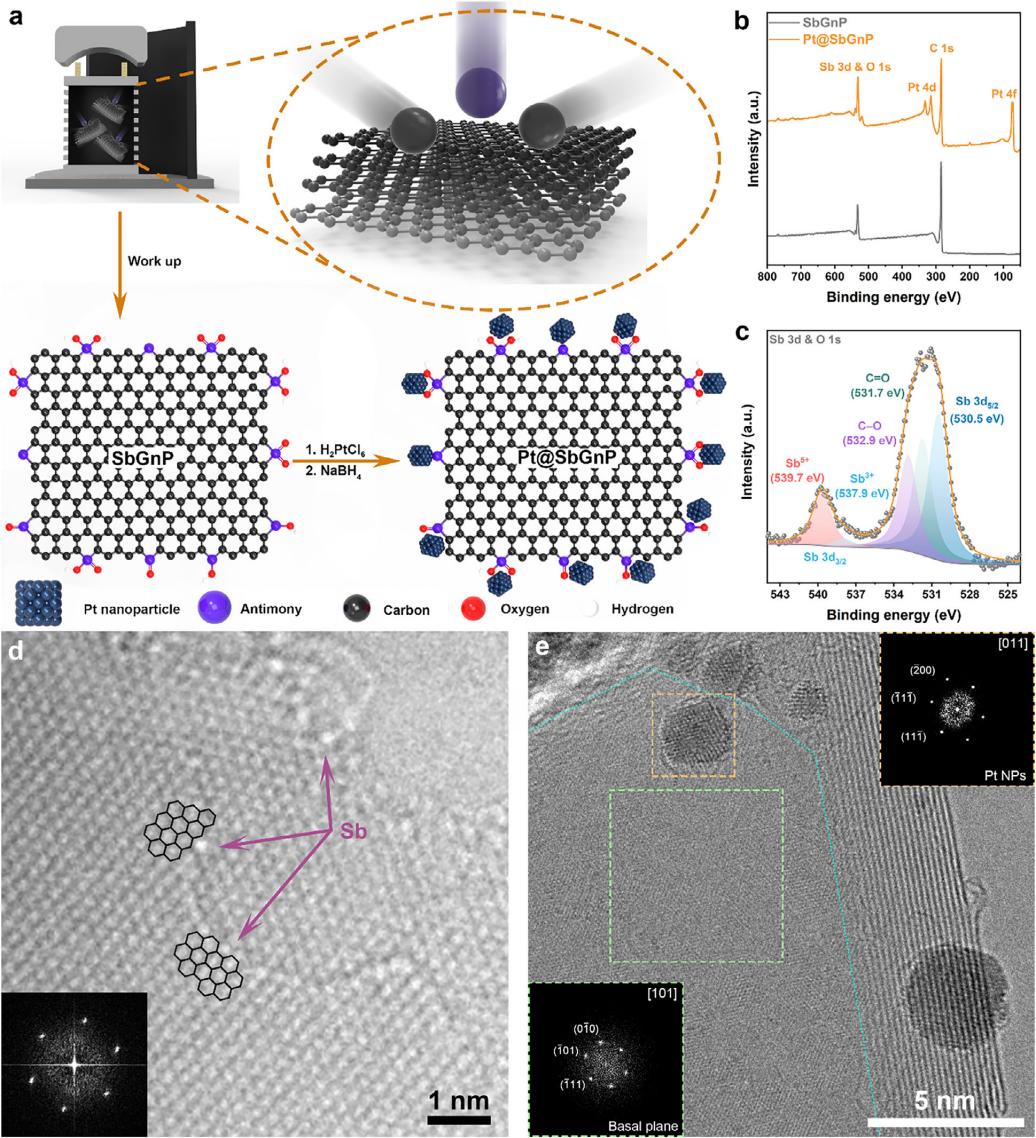
Preparation and structural analysis of Pt@SbGnP. a) Schematic illustration of the preparation of Pt@SbGnP. b) XPS survey spectra of SbGnP and Pt@SbGnP. c) Deconvolution of high‐resolution XPS spectra of Pt@SbGnP for Sb. d) AR‐TEM image of a single Sb atom attached at the edges of graphitic nanoplatelets. The inset is the corresponding fast‐Fourier transform (FFT) pattern. e) HR‐TEM image of Pt@SbGnP. The right‐up inset is the corresponding fast‐Fourier transform (FFT) pattern of the Pt nanoparticle in the orange square area, and the left‐bottom inset is the FFT pattern of a graphitic basal plane in the green square area. The cyan line separates the basal plane and edge part of Pt@SbGnP.

A schematic illustration of the preparation of Pt@SbGnP is shown in **Figure** **1**a. The metalloid Sb functionalized graphitic nanoplatelets (SbGnP) were prepared by the mechanochemical reaction of graphite and Sb ore.^[^ ^21^ ^]^ The Pt nanoparticles were deposited on the SbGnP by a conventional method to yield Pt nanoparticles on SbGnP (Pt@SbGnP).^[^ ^22^ ^]^ XRD patterns of the SbGnP and Pt@SbGnP showed a [002] diffraction peak at 2*θ* value of 24.1° (d‐spacing 0.37 nm, Figure S1, Supporting Information), which is lower than a typical graphitic [002] peak at 26.5° (d‐spacing 0.34 nm).^[^ ^23^ ^]^ This occurred because the graphitic layers were expanded by the formation of C─Sb bonds along the edges of the SbGnP. In addition to the graphitic [002] peak at 24.1°, the diffraction peaks at 2θ values of 39.7, 46.2, 67.6, and 81.6° were, respectively, assignable to [111], [200], [220], and [311] lattice planes of the face‐centered cubic (fcc) Pt crystals on Pt@SbGnP.^[^ ^24^, ^25^ ^]^ Using the Scherrer equation, the volume‐averaged size of the Pt nanoparticles on the Pt@SbGnP was calculated to be 3.7 nm.^[^ ^26^ ^]^

The chemical bonding states of SbGnP and Pt@SbGnP were confirmed using X‐ray photoelectron spectroscopy (XPS) analysis (Figure 1b). The survey spectra of both the SbGnP and Pt@SbGnP showed C 1s, O 1s, and Sb 3d peaks. In addition, Pt@SbGnP showed characteristic Pt 4d and Pt 4f peaks from the Pt nanoparticles. The high‐resolution XPS spectrum of Pt@SbGnP is shown in Figure 1c and Figure S2 (Supporting Information). The Sb 3d peaks are associated with Sb 3d_5/2_ (530.5 eV) and Sb 3d_3/2_. The Sb 3d_5/2_ are split into Sb^5+^ (539.7 eV) and Sb^3+^ (537.9 eV). Thus, the formation of a C─Sb bond of Pt@SbGnP was confirmed (Figure S2a, Supporting Information). The char yields of SbGnP and Pt@SbGnP at 1000 °C in the air were 10.7 and 24 wt.%, respectively (Figure S3, Supporting Information). Because the boiling point of Sb is as high as 1587 °C, the Pt content in Pt@SbGnP could be estimated to be 13.3 wt.% from the difference in char yields between SbGnP and Pt@SbGnP.

The morphologies of the Pt@SbGnP were investigated using scanning electron microscopy (SEM) and transmission electron microscopy (TEM). Pt@SbGnP showed a small rounded edge morphology with a grain size of less than 1 µm. Pt nanoparticles on Pt@SbGnP were uniformly distributed along the edges of the SbGnP (white dots in Figure S4, Supporting Information). We employed TEM analysis to demonstrate the C─Sb bonds along the edges of the SbGnP. Atomic‐resolution (AR)‐TEM images show that Sb atoms are present along the edges of the SbGnP (bright atomic contrast in Figure 1d). It is noteworthy that a single Sb atom is attached to the edge of graphitic layers, indicating the formation of C─Sb bonds. In the TEM image of Pt@SbGnP, Pt nanoparticles were mainly distributed at the edge sites with Sb functional groups rather than the basal plane of SbGnP (Figure 1e). This result suggests that the bond selectivity between the Sb functionalized edges and Pt nanoparticles is higher than that between a graphitic basal plane and Pt nanoparticles. Edge‐focused images further suggest that Pt nanoparticles are located along the edges of the SbGnP (Figure S5, Supporting Information). The size distribution of Pt nanoparticles is in the range of 2–5 nm, which is within the optimum size range for maximum electrocatalytic activities.^[^ ^27^, ^28^ ^]^ The fast Fourier transform (FFT) image of a Pt nanoparticle (inset image of Figure 1e) reveals high crystallinity. Its calculated lattice distance is 0.223 nm.^[^ ^29^ ^]^ In addition, the Pt@SbGnP showed a few graphitic layers with a crystalline honeycomb lattice pattern and lattice distance of 0.211 nm.^[^ ^30^ ^]^ The high crystallinity of SbGnP should be beneficial to the electrochemical kinetics. The uniform distribution of the Pt nanoparticles on SbGnP was further confirmed by scanning TEM (STEM) image, and corresponding energy‐dispersive X‐ray spectroscopy (EDS) elemental mapping images (Figure S6, Supporting Information).

**Figure 2:**
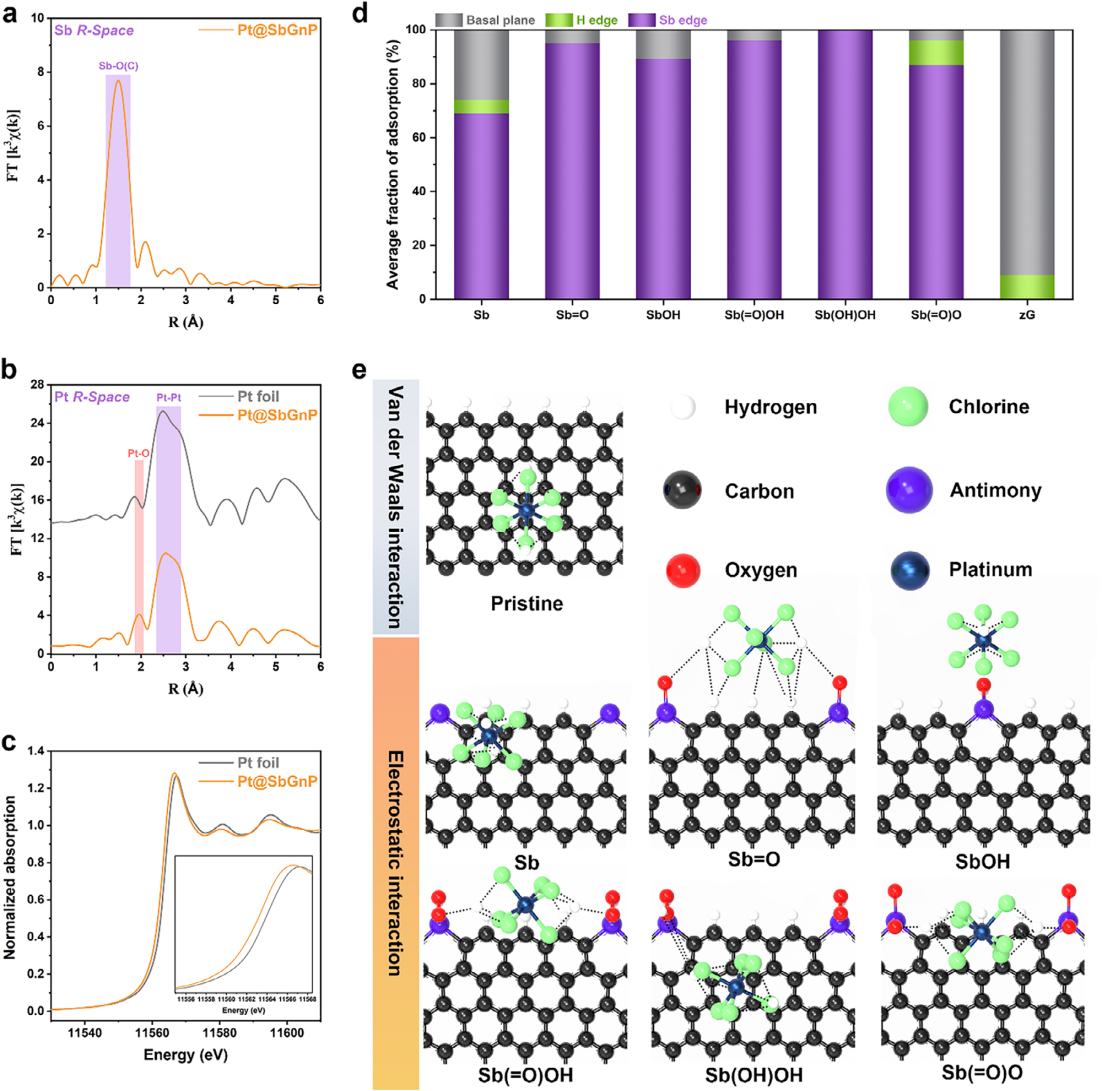
Spectroscopic analyses of Pt@SbGnP and selective adsorption of Pt precursors on Sb sites. a) EXAFS Fourier transform of k^3^−weighted Sb K−edge of Pt@SbGnP. b) EXAFS Fourier transform of k^3^−weighted Pt L_3_−edge of Pt@SbGnP and Pt foil. c) XANES spectra of the Pt L_3_−edge of Pt@SbGnP and Pt foil. d) An average of one to five PtCl_6_^2−^ ions were adsorbed to the SbGnP models. e) Stable configurations of a pair of H_2_PtCl_6_ ions adsorbed to the SbGnP models. Black dotted lines indicate closely interacting atom pairs within 3 Å.

The coordination environments and local electronic structures of the Pt@SbGnP catalysts were further characterized by X‐ray absorption spectroscopy (XAS). XAS analysis is one of the most used analytical tools to describe the metal−support interaction.^[^ ^31^, ^32^, ^33^, ^34^ ^]^ **Figure** **2**a shows the k^3^−weighted Fourier transform of the extended X‐ray adsorption fine structure (EXAFS) of the Sb K‐edge of the Pt@SbGnP catalyst, exhibiting a major peak at 1.5 Å that can be assignable to the Sb−O and Sb−C coordination. This result clearly indicated that the high‐period Sb element could form the multiple oxidation states of SbO_x_. To prove the bonding nature of Pt nanoparticles and SbGnP support, the k^3^−weighted Fourier transform of EXAFS of the Pt L_3_‐edge of the Pt@SbGnP was further investigated (Figure 2b). The Pt@SbGnP commonly showed a main peak at 2.76 Å, consistent with the Pt−Pt contribution of Pt foil. The Pt−Pt coordination number was estimated to be ≈7 for Pt@SbGnP, indicating the formation of Pt nanoparticles on SbGnP.^[^ ^35^ ^]^ In addition, the Pt@SbGnP exhibited a minor peak at 1.95 Å, which was associated with the presence of Pt−O bonds, suggesting that the bond formation between Pt nanoparticles and SbGnP support. The result supports that the Sb element has a larger atomic size with multiple oxidation states, which allows it to accommodate more chemical bonds for the formation of stable bonds with Pt nanoparticles. Thus, one part of SbGnP can be oxidized during the synthesis procedure, and it can also provide a coordination site for the stable deposition of Pt nanoparticles by forming a Pt─O bond.^[^ ^21^ ^]^ The X‐ray absorption near‐edge structure (XANES) spectra of Pt L_3_‐edge is shown in Figure 2c. The white line (WL) is related to the unoccupied density of states of the Pt 5d orbitals.^[^ ^36^ ^]^ Compared to Pt foil, the strong WL of Pt@SbGnP suggests that the Pt has increased d‐orbital vacancies and oxidation states, indicating that Pt@SbGnP forms the coordination between Pt nanoparticles and Sb functional groups, namely SbO_x_. The increased d‐orbital vacancies in Pt nanoparticles can allow an efficient electron transfer from SbGnP to Pt nanoparticles.

In order to theoretically elucidate the selective Pt precursor adsorption process of the Sb functional groups (SbO_x_) of SbGnP during the synthesis process, it was further investigated using a Monte Carlo (MC) annealing simulation. Figure 2d and Figure S7 (Supporting Information) shows the distribution of Pt precursor (i.e., PtCl_6_^2−^) adsorbed in the SbGnP model. In the pristine GnP model (“Pristine”), the Pt precursors were mainly adsorbed on the basal plane region, whereas, in the SbGnP model, Pt adsorption was more favorable at the Sb edge sites. In particular, highly selective adsorption was observed in the Sb functional group model containing both Sb(OH) and Sb(═O). From the most stable configurations of adsorption, we observed that the Pt precursors adsorbed to the basal plane of the pristine GnP model had interacted with several carbon atoms forming multiple van der Waals interactions (Figure 2e). In contrast to pristine GnP, in Pt@SbGnP, the H_2_PtCl_6_ showed stronger electrostatic interactions with one or more of the oxygen atoms attached to the Sb functional groups (SbO_x_). These results were further supported by electron density maps (Figure S8, Supporting Information). The electron density of SbGnP was much higher at the edge sites than at the basal plane, indicating that Sb functional groups could provide the binding sites for enhanced electrostatic interaction with the cationic Pt precursors. This result was well aligned with the XAS analysis.

**Figure 3:**
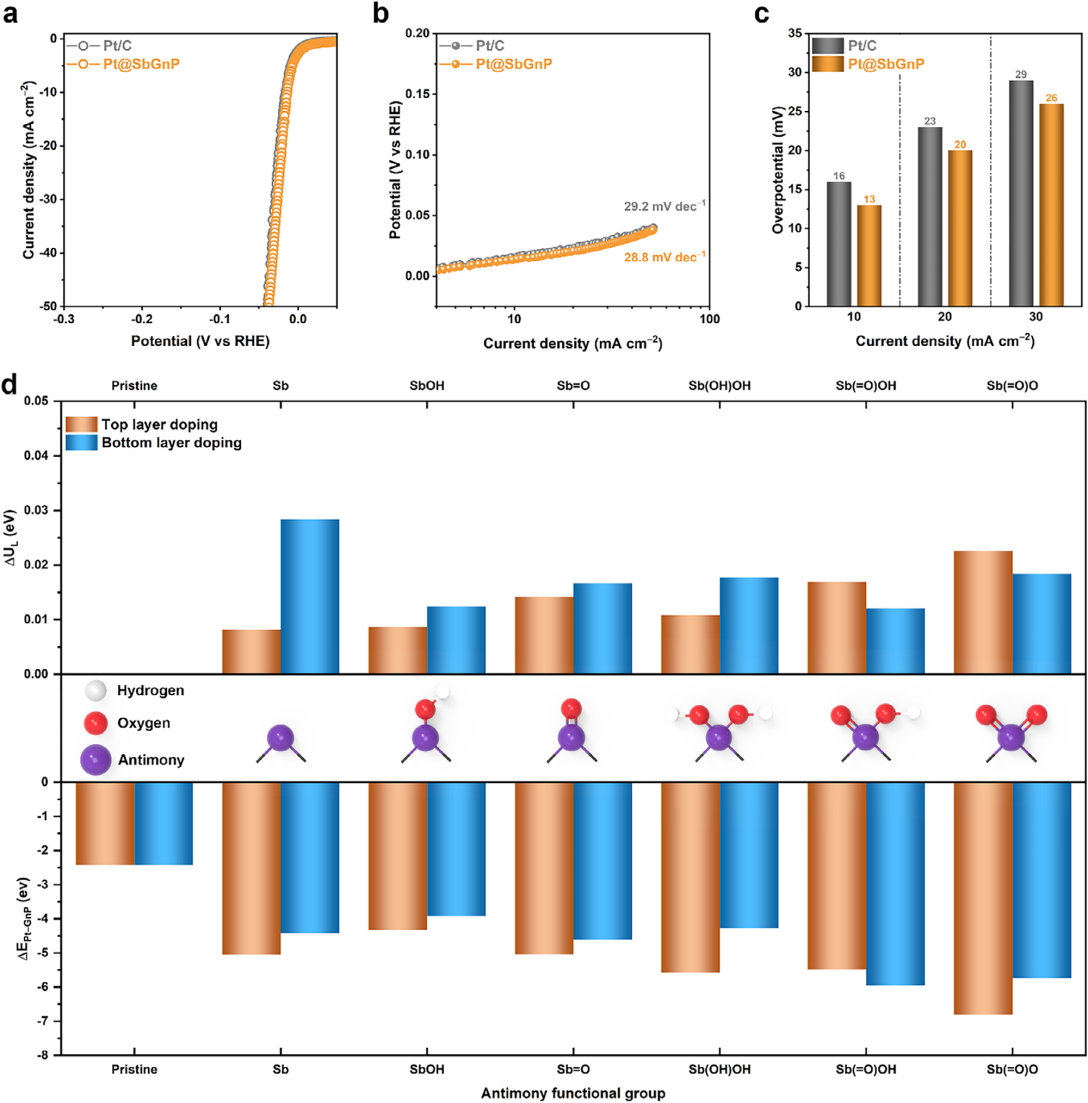
Experimental electrochemical HER performance and theoretical DFT calculations. a,b) Polarization curves and corresponding Tafel plots of the Pt@SbGnP and commercial Pt/C in Ar‐saturated 0.5 _M_ aq. H_2_SO_4_ solution. Scan rate: 5 mV s^−1^. c) Overpotentials at 10, 20, and 30 mA cm^−2^. d) Relative limiting potentials (ΔU_L_) for the HER of Pt@SbGnP models, binding energies (ΔE_Pt−GnP_) between Pt and pristine SbGnP layer of Pt@SbGnP models.

Given the unique structural information Pt@SbGnP, the electrocatalytic HER performance of Pt@SbGnP was first evaluated using a typical three‐electrode system, in Ar‐saturated 0.5 _M_ aq. H_2_SO_4_ solution. As references, SbGnP and commercial Pt/C were also tested under the same conditions. As shown in the linear sweep voltammetry (LSV) curves, SbGnP showed poor catalytic activity toward HER in the range of applied potential (Figure S9, Supporting Information). On the other hand, Pt@SbGnP showed an excellent enhancement of activity, whose onset potential approached 0 mV to induce hydrogen evolution, close to the thermodynamic potential of HER (**Figure** **3**a). The HER kinetics were evaluated by comparing the Tafel slopes, which provide mechanistic insights into electrocatalysts. The HER current densities of both Pt@SbGnP and the reference Pt/C sharply increased as the overpotential increased and their Tafel slopes are almost the same (29 mV dec^−1^) (Figure 3b), indicating that the rate‐determining step is the recombination of chemisorbed hydrogen, following the Volmer‐Tafel mechanism.^[^ ^37^, ^38^ ^]^ Pt@SbGnP required small overpotentials of 13, 20, and 26 mV to achieve current densities of 10, 20, and 30 mA cm^−2^, respectively (Figure 3c). The values are relatively smaller than those of Pt/C, indicating that the HER polarization curve of Pt@SbGnP with lower Pt content (13.3 wt.%) has more positive shifted overpotential than the Pt/C with higher Pt content (20 wt.%).

To demonstrate the reduced HER overpotential of Pt@SbGnP, the theoretical limiting potential (ΔU_L_) for various antimony functional groups was evaluated using density functional theory (DFT) calculations. The relative limiting potentials (ΔU_L_) of various antimony functional groups against pristine are shown in Figure 3d. The ΔU_L_ of HER was estimated to be <0.03 eV. This positive value is consistent with the experimental trend of the positive shift of HER overpotential. In particular, the Sb(═O)O group on the top layer and the Sb group on the bottom layer increased the limiting potential by more than 0.02 eV. Overall, the Sb functional groups played a positive role, in improving HER activity. The introduction of Sb functional groups can contribute to Pt sites, reducing overpotential and thus enhancing HER catalytic activity.

The electrochemical impedance spectroscopy (EIS) analysis of Pt@SbGnP exhibited charge transfer resistances of 0.71, 0.62, and 0.5 Ω cm^2^ at overpotentials of 10, 15, and 20 mV (Figure S10, Supporting Information). These values are much lower than those of Pt/C. The results indicate there was faster electron/proton transfer at the interface of Pt@SbGnP and the electrolyte. Next, the electrochemically active surface area (ECSA), which is proportional to the double‐layer capacitance (C_dl_) of the electrocatalyst, was tested (Figure S11, Supporting Information). The C_dl_ of Pt@SbGnP (72.2 mF cm^−2^) was considerably larger than that of the Pt/C (21.7 mF cm^−2^), indicating that the ECSA of Pt@SbGnP was extremely expanded, providing more active sites to enhance HER activity. To quantitatively analyze the number of active sites, which is one of the most important factors for enhancing HER activity, along with the expanded ECSA, a copper under‐potential deposition (Cu‐UPD) of Pt@SbGnP was performed (Figure S12, Supporting Information). The active sites in Pt@SbGnP were determined to be 16.66 × 10^−4^ mol g_pt_^−1^, which is much higher than Pt/C (10.47 × 10^−4^ mol g_pt_^−1^). We further evaluated turnover frequency (TOF), which is an important criterion for HER catalysts, to determine intrinsic electrocatalytic efficiency.^[^ ^39^ ^]^ The TOF value of Pt@SbGnP at 20 mV was 0.68 H_2_ S^−1^, which is higher than Pt/C (Figure S13, Supporting Information).

To interpret the active sites on Pt@SbGnP, thiocyanate ion (^–^SCN), a toxin to active sites on metal catalysts, was added to 0.5 _M_ aq. H_2_SO_4_ electrolyte. When ^–^SCN (5 mm) was introduced into the acidic electrolyte, the overpotential of Pt@SbGnP at a current intensity of 10 mA cm^−2^ increased from 13 (before) to 133 mV (after), because ^−^SCN dramatically reduced the active sites on the Pt@SbGnP. The result demonstrated that the Pt nanoparticles on Pt@SbGnP act as active sites for HER (Figure S14, Supporting Information).

For a fair comparison of catalytic activity, the polarization curves of Pt@SbGnP and Pt/C were normalized by ECSA, and the loading amounts of Pt were determined (Figure S15, Supporting Information). In 0.5 _M_ aq. H_2_SO_4_ solution for a series of overpotentials, Pt@SbGnP showed slightly higher specific activity than Pt/C. At an overpotential of 20 mV, the specific activity of Pt@SbGnP was 0.2 mA cm^−2^, which was higher than Pt/C (Figure S15a, Supporting Information). This result indicates that Pt@SbGnP possesses superior intrinsic catalytic activity, which can be attributable to an optimized hydrogen adsorption behavior on the surface of Pt nanoparticles.^[^ ^40^ ^]^

As a result, Pt@SbGnP can supply fast protons for more efficient hydrogen generation. To further examine the catalysts from different perspectives, the mass activity of each catalyst was evaluated by normalizing the polarization curves according to Pt mass, because it is closely related to cost in practical applications. At an overpotential of 20 mV in acidic conditions, the mass activity of Pt@SbGnP was 205.9 mA mg_pt_^−1^ (Figure S15b, Supporting Information), which was over twice as high as Pt/C (107.7 mA mg_pt_^−1^). Therefore, it can be stated that Pt@SbGnP has significant advantages over the commercial Pt/C in terms of its overall catalytic performance and cost.

To compare the long‐term stability of Pt@SbGnP and Pt/C, cyclic stability tests were conducted in 0.5 _M_ aq. H_2_SO_4_ at a scan rate of 100 mV s^−1^ (Figure S16, Supporting Information). After 10 000 cycles, Pt@SbGnP and Pt/C showed a negative shift of 6 and 10 mV, respectively, at a current density of 10 mA cm^−2^. Notably, the amount of change in the overpotential of Pt@SbGnP was smaller than Pt/C at a higher current density (from 10 to 50 mA cm^−2^). Stability was also examined via the current–time (I–t) test at a constant potential of 15 mV in acidic conditions for 50 h. Pt@SbGnP exhibited no apparent loss in current density compared to Pt/C.

In order to confirm the improved stability, an additional proof of interaction between the Pt nanoparticles and SbGnP was carried out. The aggregation of Pt nanoparticles was dramatically reduced by the introduction of Sb functional groups on GnP (SbGnP), implying that the interaction between Pt nanoparticles and SbGnP was improved. To further explain the origin of this enhanced stability, the interactions between the Pt nanoparticles and Sb functional groups on Pt@SbGnP were estimated using DFT calculations. The results showed that ΔE_Pt−GnP_ was significantly improved, from −2.43 to −3.61–−6.80 eV when Sb functional groups were introduced in the GnP (Figure 3d). Single Sb atoms at the edges of the SbGnP significantly increased the interaction with the Pt nanoparticles, and oxygenated groups [Sb(OH)OH and Sb(═O)OH] on the Sb atoms further improved the interactions. These results indicate that both Pt─Sb and Pt─O bonds formed at the interface of the Pt nanoparticles and the SbGnP in Pt@SbGnP played an important role in improving the interaction. As shown in Figure S17 (Supporting Information), the Sb and O atoms of the Sb functional groups on the top layer were simultaneously coordinated with Pt nanoparticles. However, when Sb was functionalized on the bottom layer, the interactions were weakened, due to the longer distance Pt─Sb bonds, while Pt−O interactions remained largely uninfluenced by the location of the Sb functional groups. Clearly, the existence of Sb functional groups improved the interaction between Pt nanoparticles and SbGnP, accounting for the reduced aggregation of Pt nanoparticles. Both experimental and DFT calculation results suggested that the improved HER stability of Pt@SbGnP originated from the improved interaction between the Sb functional groups and Pt nanoparticles.

**Figure 4:**
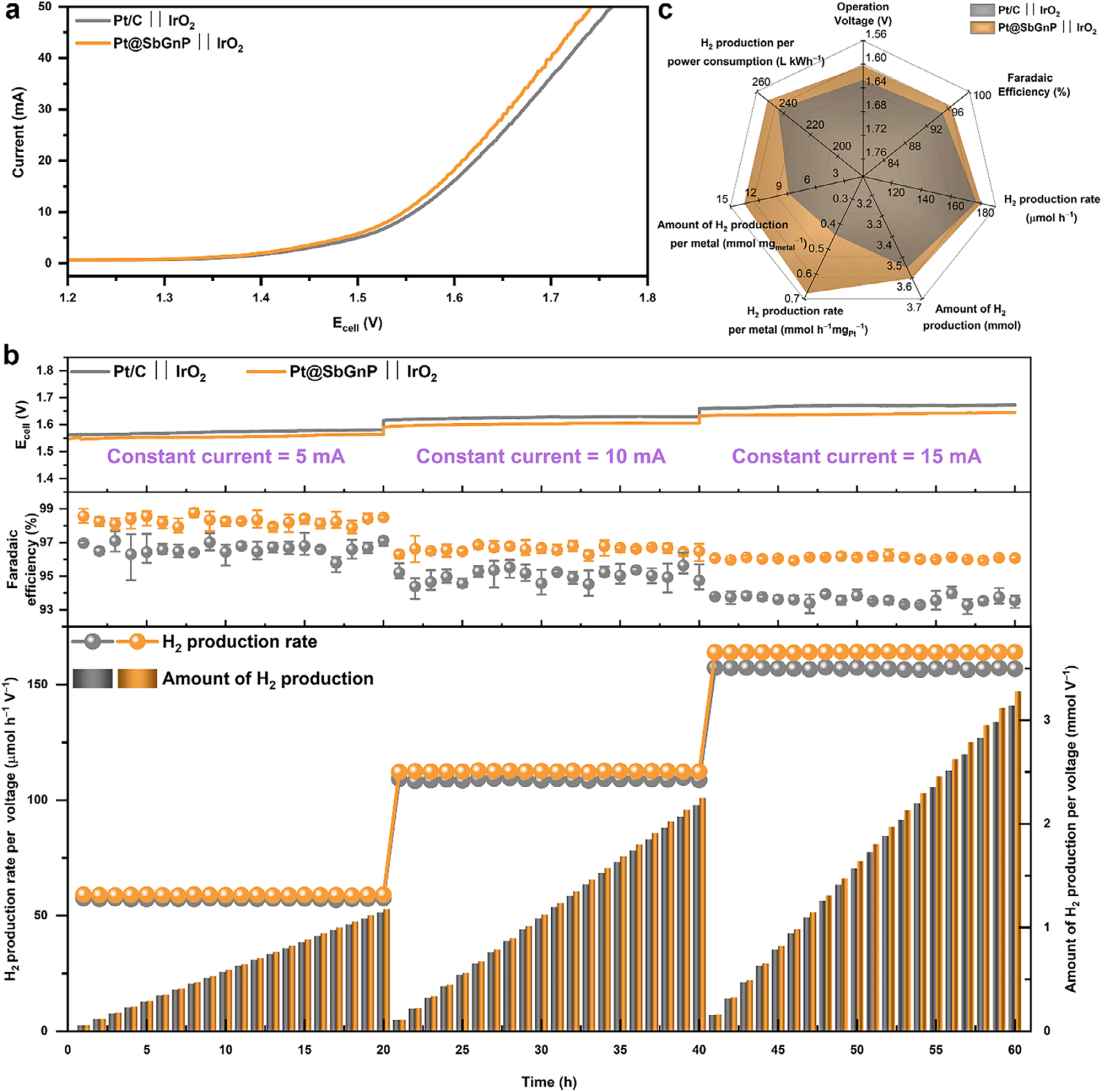
Full water splitting evaluation. a) Polarization curves in Ar‐saturated 0.5 _M_ aq. H_2_SO_4_ solution. Scan rate: 5 mV s^−1^. b) Cell potential changes at constant currents, Faradaic efficiency, hydrogen production rate, and amount of hydrogen production per voltage at specific currents of 5, 10, and 15 mA. The error bars reflect the three device results. c) Comparison of radar chart of performance factors between the Pt@SbGnP and commercial Pt/C‐based water electrolyzer systems.

Inspired by the high catalytic activity of Pt@SbGnP in the half‐cell test, a two‐electrode conventional electrolyzer system was assembled to evaluate the full water splitting performance, using commercial iridium oxide (IrO_2_) as the anode (Figure S18, Supporting Information). The cathode and anode electrodes were prepared by coating the catalyst on carbon paper using the e‐spray method. As illustrated in **Figure** **4**a, the Pt@SbGnP also exhibited high water‐splitting performance in a conventional electrolyzer. To deliver currents of 5, 10, and 15 mA, the Pt@SbGnP required cell voltages of 1.56, 1.60, and 1.64 V, respectively, lower than that of Pt/C (Figure 4b). The hydrogen production rates of Pt@SbGnP were ≈91.7, 180.2, and 268.9 µmol h^−1^ at 5, 10, and 15 mA, respectively, surpassing those of Pt/C. The total amounts of hydrogen generated during 20 h were 1.8, 3.6, and 5.4 mmol at 5, 10, and 15 mA, respectively. The values were ≈1.4 to 2.7% larger than Pt/C (Figure S19 and Tables S3 and S4, Supporting Information). More importantly, the Pt utilization of Pt@SbGnP was significantly higher than that of Pt/C (Figure S20, Supporting Information). The rate of hydrogen production per platinum content of Pt@SbGnP was 0.34, 0.68, and 1.01 mmol h^−1^mg_pt_^−1^ at 5, 10, and 15 mA, which was faster than those of Pt/C. In addition, after 20 h, the amount of hydrogen production per platinum content was 20.22 mmol mg_pt_^−1^, which is 1.5 times more than Pt/C (Figure S21, Supporting Information). These results clearly show that Pt@SbGnP can maximize Pt activity on HER compared to the commercial Pt/C. Therefore, the Pt@SbGnP is a cost‐competitive catalyst for HER. Faradaic efficiency, which represents the electron utilization efficiency, is an important parameter for catalytic performance. That is, the Faradaic efficiency approaches 100% as the electron utilization efficiency for the HER grows higher. The Faraday efficiencies for Pt@SbGnP were 98.29, 96.61 and 96.08% at 5, 10, and 15 mA, which were higher than Pt/C, indicating that the Pt@SbGnP efficiently utilized electricity for HER (Figure S22a and Table S5, Supporting Information). From the viewpoint of commercialization, the values of hydrogen production per power consumption for Pt@SbGnP were calculated to be ≈264.0, 252.0, and 245.0 L kWh^−1^ at 5, 10, and 15 mA, respectively (Figure S22b and Table S6, Supporting Information). If we take account of the catalyst cost, those values can be further normalized by the Pt content. The values of hydrogen production per power consumption and Pt content were evaluated to be ≈992.4, 947.2, and 920.9 L kWh^−1^ mg_pt_^−1^, which were much higher than those of Pt/C. All comparison factors suggest that the Pt@SbGnP is better than Pt/C, especially in terms of mass activity (Figure 4c). Therefore, it is clear that the Pt@SbGnP is a cost‐efficient catalyst. With the much lower Pt usage, the production cost of H_2_ on Pt@SbGnP was estimated to be ≈3.02 USD kg^−1^, and very close to the US Department of Energy (DOE) goal by 2026 (2 USD kg^−1^), based on the business electricity price of South Korea (0.071 USD kWh^−1^).

**Figure 5:**
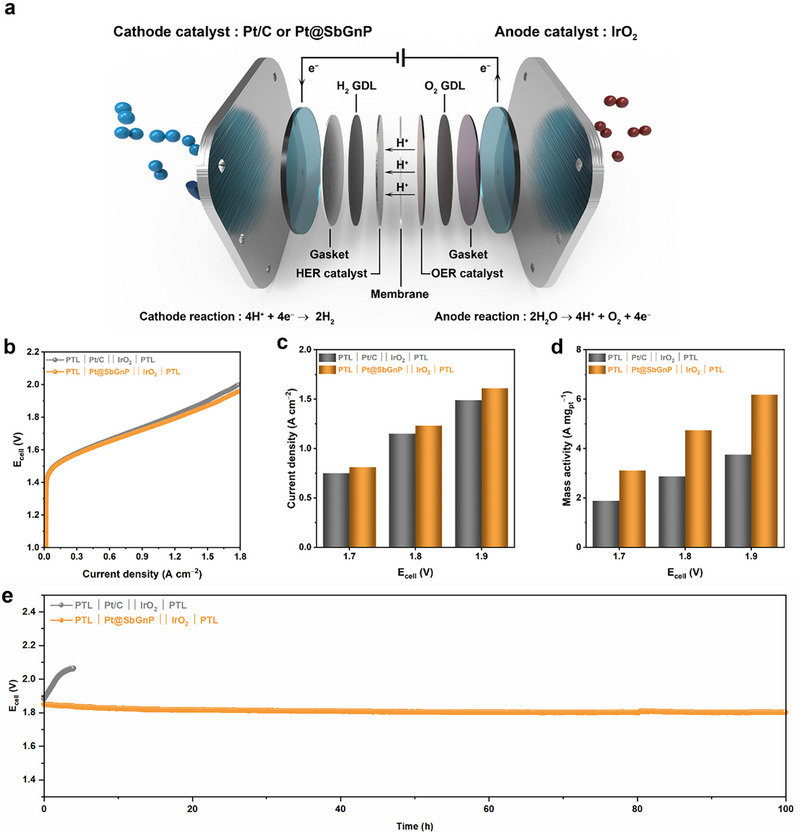
Single‐cell performances of water electrolyzer. a) Schematic diagram of a water electrolyzer configuration. b) Polarization curves of water electrolyzer. c) Current densities at different operating voltages. d) Mass activities at different operating voltages. e) Stability test at 1A cm^−2^.

To test the possibility of implementing Pt@SbGnP in a practical water electrolyzer device, single‐cell performances were evaluated using a membrane electrode assembly (MEA) (**Figure** **5**a). Measurements were made in the system using a peristaltic pump for electrolyte circulation and the separation of hydrogen and oxygen gases (Figure S23, Supporting Information). The MEAs with Pt/C and Pt@SbGnP as the cathodes showed a similar onset cell potential at 1.5 V (Figure 5b). However, at 1.8 V, the Pt@SbGnP current showed density values of 1.23 A cm^−2^, outperforming the commercial Pt/C with 1.15 A cm^−2^ (Figure 5c). The mass activity of Pt@SbGnP was 4.74 A mg_pt_^−1^, while that of the commercial Pt/C was only 2.87 A mg_pt_^−1^ at 1.8 V (Figure 5d). Durability tests were conducted at 1 A cm^−2^ and 80 °C, revealing a rapid activity decay of the commercial Pt/C catalyst within 4 h, while the Pt@SbGnP exhibited high stability without noticeable decay for 100 h (Figure 5e). After the durability tests, TEM analysis revealed notable differences between samples (Figure S24, Supporting Information). While a severe aggregation of Pt nanoparticles was observed in Pt/C, Pt@SbGnP maintained a uniform Pt nanoparticle distribution, suggesting the superior durability of Pt@SbGnP, which could be attributed to the SbGnP support. Additionally, XPS analysis conducted after the durability tests confirmed the stability of Pt@SbGnP (Figure S25, Supporting Information). The Pt 4f spectra showed minimal shifts in binding energy, indicating that the electronic structure of Pt remained largely unchanged after prolonged operation. This suggests that the heteroatom‐doped carbon support effectively stabilizes Pt nanoparticles, preventing oxidation or leaching under harsh electrochemical conditions.

## 3 Conclusion

In summary, an efficient and stable electrocatalyst for HER was developed via simple cost‐effective procedures, including mechanochemical and solvothermal reactions. The catalyst consisted of Pt nanoparticles anchored on metalloid antimony (Sb) functionalized graphitic nanoplatelet (Pt@SbGnP). As a catalytic support, the SbGnP not only provided SbO_x_ sites for stably anchoring Pt nanoparticles for improved catalytic activity and durability, but also, in addition to its high crystallinity, modulated electronic structures of Pt nanoparticles for efficient electron transfer. The Pt@SbGnP was systematically evaluated as an electrocatalyst for the hydrogen evolution reaction (HER), based on various contributing factors, including overpotential, charge transfer resistance, active sites, mass, and specific activity. The overall electrocatalytic performance of the Pt@SbGnP was superior to the state‐of‐the‐art Pt/C. Further experiments with an actual water‐splitting system demonstrated its strong potential for practical applications. Most importantly, Pt@SbGnP could be mass‐produced at low cost, satisfying all three requirements (efficiency, stability, and cost) for commercial hydrogen production. Finally, further research can be directed toward establishing a correlation between structure and electrocatalytic performance with respect to high‐period “metalloid” functional groups (e.g., arsenic, tellurium, and so on) rather than typical nonmetallic elements (e.g., N, O, P, S, and so on). The findings in this work can provide a variety of new insights to further develop high‐performance electrocatalysts.

## 4 Experimental Section

### Materials

Graphite was obtained from Alfa Aesar (Natural, 100 mesh, 99.9995% metals basis, Lot #: 14 735) used as received. Antimony (Sb) was purchased from Aldrich Chemical Inc. (Cat.# 266 329) and used as received. All other solvents were supplied by Aldrich Chemical Inc. and used without further purification unless otherwise specified.

### Preparation of SbGnP

SbGnPs were prepared by ball‐milling graphite in the presence of Sb ore in a planetary ball‐mill capsule.^[^ ^21^ ^]^ Graphite (5.0 g) and Sb (5.0 g) were placed into a stainless‐steel capsule containing stainless‐steel balls (500 g, diameter 5 mm). After the capsule was sealed, five cycles of charging (70 psi) of argon and discharging at reduced pressure (0.05 mm Hg) were conducted to completely remove air. The capsule was then fixed in the planetary ball‐mill machine, and agitated at 500 r.p.m. for 48 h. The resultant product was repetitively washed off with warm concentrated HCl (≈37%) to remove remaining metallic impurities and unreacted Sb, if any. The final product was Soxhlet extracted with concentrated HCl and then freeze‐dried at 120 °C under reduced pressure (0.05 mm Hg) for 48 h to yield a dark black SbGnP powder.

### Preparation of Pt@SbGnP

In a one‐necked round bottom flask, SbGnP (200 mg) was dispersed in de‐ionized water (100 mL) by sonication for 1 h. Chloroplatinic acid hydrate (H_2_PtCl_6_·H_2_O, 50 mg) in de‐ionized water (50 mL) was added to the reaction mixture and vigorously stirred for 24 h. Pt nanoparticles were formed by adding sodium borohydride (NaBH_4_, 0.1 m aq. solution, 100 mL) while vigorously stirring. After the completion of the reaction, Pt nanoparticles on SbGnP (Pt@SbGnP) were collected by filtration, washed with de‐ionized water several times, and freeze‐dried at −120 °C under reduced pressure (0.05 mm Hg) for 48 h to yield Pt@SbGnP (216 mg).

### Electrochemical Characterizations

The electrochemical studies were conducted on an electrochemical workstation (Ivium, Netherlands) with a typical three‐electrode cell. A graphite rod and an Ag/AgCl (saturated KCl) electrode were used as the counter electrode and reference electrode, respectively. All the potentials were referenced with a reversible hydrogen electrode (RHE). The catalysts (5 mg) and Nafion (20 µL, 5 wt.% in a mixture of lower aliphatic alcohol and water, Aldrich Chemical Inc.) were dispersed in 1.0 mL isopropyl alcohol solution, followed by ultrasonication for 30 min under the ice bath condition to form uniform catalyst inks. The inks were dropped onto rotating ring‐disk electrodes (4 mm in diameter) to form a film for the electrochemical tests. The loading amounts of the catalysts were 0.70 mg cm^−2^. Linear sweep voltammetry was conducted in 0.5 _M_ aq. H_2_SO_4_ solution with a scan rate of 5 mV s^−1^. The solution resistances (R_s_) of 6 Ω in the 0.5 _M_ aq. H_2_SO_4_ solution were tested by Nyquist plots, which were further used for the ohmic drop (iR) correction of all the HER data (Figure S26, Supporting Information). The reference electrode was calibrated, and all potentials were referenced to a RHE (Figure S27, Supporting Information).

### Electrochemical Active Surface Area Calculations

The effective active surface area of the sample was determined according to the literature method.^[^ ^38^ ^]^ Cyclic voltammetry (*C–V*) was carried out at different scan rates versus the RHE region. The double‐layer capacitances (C_dl_) of different samples were determined from the cyclic voltamograms. The C_dl_ was proportional to the electrochemical surface area of the sample. Therefore, the high values of C_dl_ for Pt@SbGnP were because of the small size of the Pt nanoparticles (≈3.7 nm) and the high specific surface area provided by the SbGnP matrix.^[^ ^21^ ^]^ To calculate electrochemical capacitance, the potential was swept five times from 0.10 to 0.30 V (versus RHE) for HER at each of eleven different scan rates (10, 20, 40, 50, 60, 80, and 100 mV s^−1^). Capacitive currents were determined in a potential range where no Faradic processes were observed. The difference (∆J) in measured capacitive currents was plotted as a function of scan rate in Figure S11 (Supporting Information). Specific capacitance was measured from the slope of the linear fitting. The specific capacitance was transformed into an electrochemical active surface area (ECSA) using the specific capacitance value for a flat standard with 1 cm^2^ of real surface area.^[^ ^41^ ^]^ The specific capacitance for a flat surface was generally found to be in the range of 20–60 µF cm^−2^.^[^ ^41^ ^]^ In the following calculations, it was assumed to be 40 µF cm^−2^. In this work, ECSA calculations were performed using the following equation.

(1) $$A_{ECSA}^{Sample}=\frac{C_{dl}\cdot mF\cdot cm^{-2}}{40\mu F\cdot cm^{-2}percm_{ECSA}^{2}}=\left(value\right)cm_{ECSA}^{2}$$

### Active Sites Calculations

The underpotential deposition (UPD) of copper (Cu) was used to calculate the active sites of the Pt@SbGnP and Pt/C. In this method, the number of active sites (n) can be calculated based on the UPD copper stripping charge (Q_Cu_, Cu_upd_ → Cu^2+^ + 2e^−^) using the following equation.^[^ ^38^ ^]^

(2) $$n=Q_{Cu}/2F$$

where F is the Faraday constant (96485.3 C mol^−1^).

### Measurement of the Turnover Frequency (TOF)

The TOF (s^−1^) was calculated with the following equation.

(3) $$\mathrm{TOF}=I/\left(2\mathrm{Fn}\right)$$

where I is the current (A) during linear sweep voltammetry (LSV), F is the Faraday constant (96 485.3 C mol^−1^), and n is the number of active sites (mol). The factor 1/2 was based on the assumption that two electrons were necessary to form hydrogen molecules.

### Preparation of Electrodes

Homogeneously dispersed sample powders (5 mg each, Pt@SbGnP, Pt/C, and IrO_2_) in 2‐propanol solution (1 mL) were obtained by sonication for 30 min. The resultant solution was deposited directly onto carbon paper using an electrospray method. First, the sample dispersion solutions were loaded into a plastic syringe equipped with a 30‐gauge stainless steel hypodermic needle. The needle was connected to a high‐voltage power supply (ESN‐HV30). A voltage of ≈4.5 kV was applied between a metal orifice and the conducting substrate at a distance of 8 cm. The feed rate was controlled by a syringe pump (KD Scientific Model 220) at a constant flow rate of 20 µL min^−1^. The electric field overcomes the surface tension of the droplets, resulting in the minimization of numerous charged mists. Each electrode was measured after drying in a vacuum for 1 day at room temperature.

### Pre‐Treatment of Nafion (212) Membrane

First, a Nafion membrane was treated with nearly boiling (≈80 °C) 3% aq. H_2_O_2_ for 2 h. Then, the membrane was immersed in nearly boiling (≈80 °C) de‐ionized water for 2 h. Finally, the membrane was treated with nearly boiling (≈80 °C) 0.5 _M_ aq. H_2_SO_4_ and subsequently rinsed with ultrapure water. After the pretreatment, the membrane was stored in de‐ionized water.

### Fabrication of Membrane Electrode Assembly (MEA)

Catalysts were coated by the substrate method to prepare the MEA. For the cathode and anode electrodes, commercial Pt/C, Pt@SbGnP, and IrO_2_ (1 mg cm^−2^ each) were used. A membrane was inserted between the anode and the cathode, and hot pressing (2 MPa) was applied at 120 °C for 3 min. Then, the MEA was mounted into homemade electrolyzer hardware with a 2 cm^2^ water flow field and a pair of platinized titanium mesh as both the anode and cathode gas diffusion layers. Finally, an end plate and PTFE gasket were used for mechanical support. The active area of a single cell was 2 cm^2^ (16 mm in diameter).

### Molecular Modeling

Pt@SbGnP models were described by a periodic three‐layered Pt slab and four‐layered zigzag graphene nanoribbon (zGNR), where Sb dopant (or oxidized Sb) was introduced (Figure S28, Supporting Information). The zGNR was set to be periodic along the *y*‐axis, and the Pt slab was placed perpendicular to the *x*‐axis interacting with one side of the zGNR. In the zGNR, two vertical layers were alternatively stacked. Two configurations were consideresd, with Sb functionalization by “Top layer doping,” where the Sb dopants were located at the outer edges closer to the Pt slab, and “Bottom layer doping,” where the Sb dopants were located at the inner edges relatively far from the Pt slab. OH and O functional groups were introduced to consider various oxidation states of the Sb dopant (i.e., Sb, Sb‐OH, Sb═O, Sb(OH)OH, Sb(═O)OHO, and Sb(═O)O). Since the Pt nanoparticles were distributed on the SbGnP, the lattice parameter of the Pt slab was fitted to that of the zGNR (i.e., 39.67 × 4.93 × 13.81 Å^3^). The lattice mismatches between the Pt slab and zGNR were 2.8 and 0.3% for the *y*‐ and *z*‐axis, respectively. For the surface of the Pt slab, the surface configuration of 100% H coverage was considered for the H adsorbed intermediate during HER.^[^ ^42^, ^43^ ^]^

### Simulated Annealing Monte Carlo (MC)

In order to observe the edge‐selective adsorption of Pt nanoparticles to the Sb‐functionalized GnP (SbGnP), Monte Carlo (MC) simulations using the simulated annealing method were carried out using the Adsorption Locator program (Materials Studio 2019; BIOVIA, Dassault Systèmes: San Diego, 2018). In the MC simulation, a monolayer zGNR system containing each type of Sb dopant was used. To remove self‐interaction with the neighboring simulation box, the DFT‐optimized unit cell structures were extended to a 1 × 1 × 5 supercell, and vacuum regions were enlarged so that the lattice parameters of the simulation box became 60 Å (Figure S29, Supporting Information). Considering the experimental synthesis process, the precursor of Pt (i.e., H_2_PtCl_6_) was treated as an ionic compound, thus H^+^ and PtCl_6_^2−^ ions were modeled with a 2:1 ratio. Atomic charges were estimated by Bader charge analysis on the Sb‐containing single‐layer zGNR via DFT calculations.^[^ ^44^, ^45^ ^]^ Nonbonding potential energy parameters between the Pt precursor ions and SbGnP were described by Universal forcefield.^[^ ^46^ ^]^ In the MC simulation, the simulated annealing run with 5 × 10^6^ MC steps for five consecutive annealing cycles was used for the screening of stable adsorption sites in the SbGnP model. The number of adsorbing Pt precursors was set to be 1 to 5 H_2_PtCl_6_ pairs to observe the adsorption process.

### Density Functional Theory (DFT) Calculations

All spin‐polarized density functional theory (DFT) calculations were conducted using the Vienna ab initio simulation package (VASP).^[^ ^47^, ^48^ ^]^ The projector‐augmented wave (PAW) pseudopotentials were used while the plane wave cutoff energy was set to 520 eV. The exchange‐correlation energy was described by the generalized gradient approximation (GGA) with the Perdew–Burke–Ernzerhof (PBE) functional.^[^ ^49^ ^]^ The DFT‐D_3_ method was applied to describe the van der Waals (vdW) interaction between the Pt slab and SbGnP layer.^[^ ^50^ ^]^ All of the considered structures were relaxed until the self‐consistent forces became smaller than 0.02 eV Å^−1^ and the total energy was changed within 10^−6^ eV per atom. A gamma‐centered k‐point mesh was sampled using the actual spacing of 0.04 Å^−1^. A vacuum slab of ≈20 Å was introduced to avoid self‐interactions. During the structural relaxation, around half of the SbGnP layer was constrained to describe the bulk SbGnP region. The limiting potentials for HER of the Pt@SbGnP models were estimated using the energy states of the reaction intermediates.^[^ ^42^, ^51^ ^]^

### Materials Characterizations

The morphologies of the samples were studied by field emission scanning electron microscopy (FE‐SEM, Nanonova 230, FEI, USA) and high‐resolution transmission electron microscopy (HR‐TEM, JEM‐2100F, JEOL, Japan). Thermogravimetric analysis (TGA) was performed at a ramping rate of 10 °C min^−1^ in air on a thermogravimetric analyzer (Q200, TA, USA). X‐ray diffraction (XRD) patterns were recorded on a high‐power X‐ray diffractometer (D/MAZX 2500 V/PC, Rigaku, Japan), using Cu‐Kα radiation (35 kV, 20 mA, λ = 1.5418 Å). An X‐ray photoelectron spectrometer (XPS, K‐alpha, Thermo Fisher Scientific, UK) and elemental analysis (EA, Flash 2000 Analyzer) were employed to determine chemical composition. The electrochemical HER test was initiated, and the evolved hydrogen gas was analyzed by gas chromatography (GC‐2010 Plus, Shimadzu, Japan), with a thermal conductivity detector (TCD). Argon was used as the carrier gas. X‐ray absorption fine spectra of the prepared catalysts were collected in the transmission mode using ionization detectors (Oxford) at the Pohang Accelerator Laboratory (PAL). The X‐ray absorption spectra for the Pt L‐edge were acquired at room temperature using beamline 6D of PAL, where the X‐ray energies from the EXAFS analysis were calibrated with Pt foil. Background subtraction, normalization, and Fourier transformation (FT) were performed using standard procedures with the ATHENA program.

## Conflict of Interest

The authors declare no conflict of interest.

## Supporting information



Supporting Information

## Data Availability

The data that support the findings of this study are available from the corresponding author upon reasonable request.
