# Supplementary material for: Platinum Nanoparticles on Metalloid Antimony Functionalized Graphitic Nanoplatelets for Enhanced Water Electrolysis
Source: Small. 2025 May 9;21(25):2501408. doi: 10.1002/smll.202501408 (PMC12199127; doi:10.1002/smll.202501408)
Supplement: Supplementary file 1 — Supporting Information [file SMLL-21-2501408-s001.docx]

Supporting Information

**Platinum nanoparticles on metalloid antimony functionalized graphitic nanoplatelets for enhanced water electrolysis**

*Do Hyung Kweon^#^, Jae-Hoon Baek^#^, Sung O Park^#^, Hyuk-Jun Noh, Jong-Pil Jeon, Jeong Hyeon Lee, Tae Joo Shin, Sang Kyu Kwak*, In-Yup Jeon*, and Jong-Beom Baek**


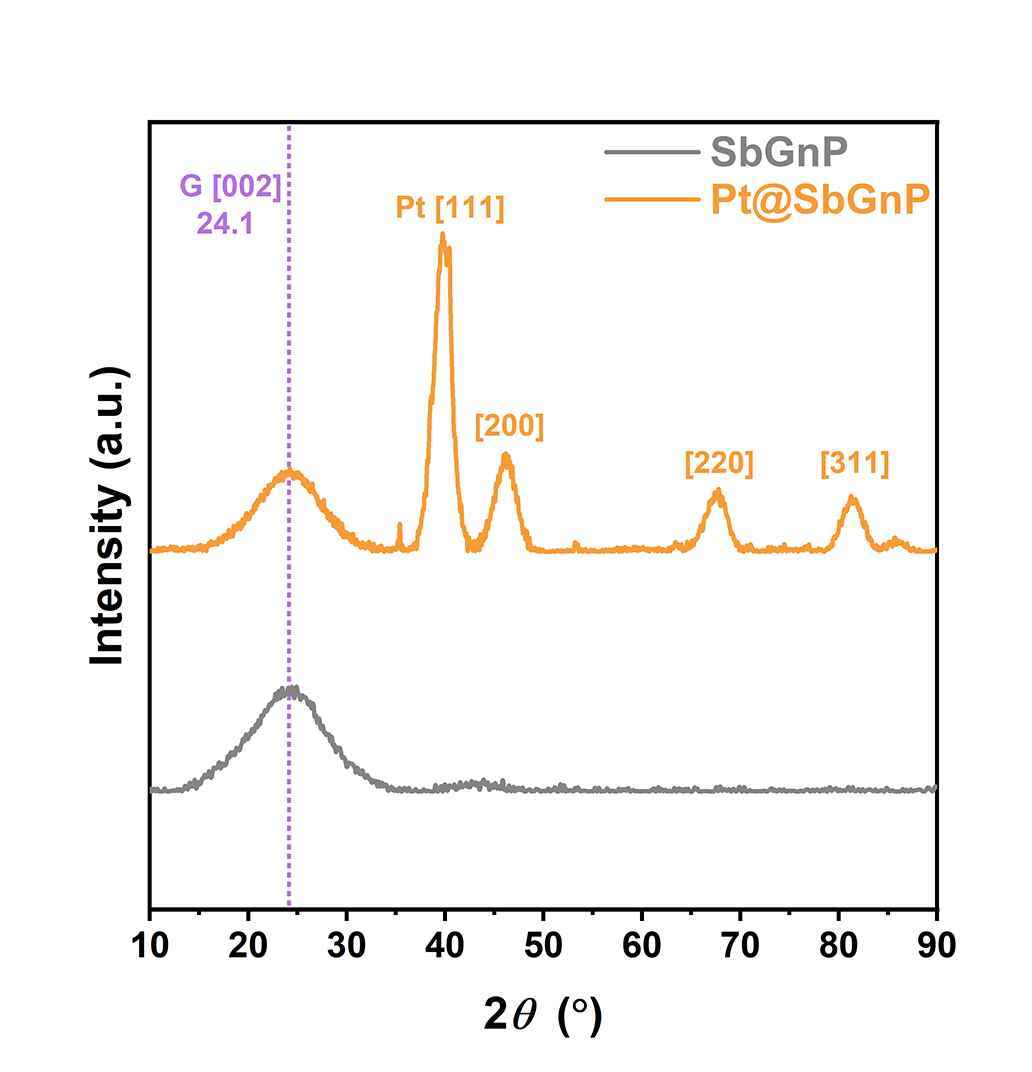


**Figure S1.** Structure characterization of SbGnP and Pt@SbGnP. Powder XRD patterns of SbGnP and Pt@SbGnP.


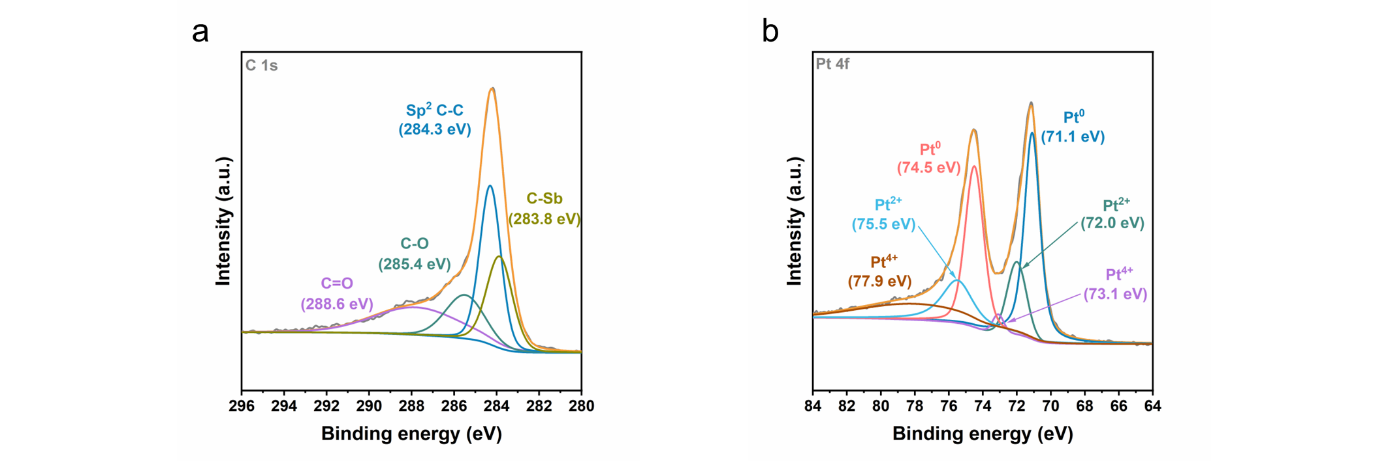


**Figure S2.** High-resolution XPS spectra: a) C 1s; b) Pt 4f.


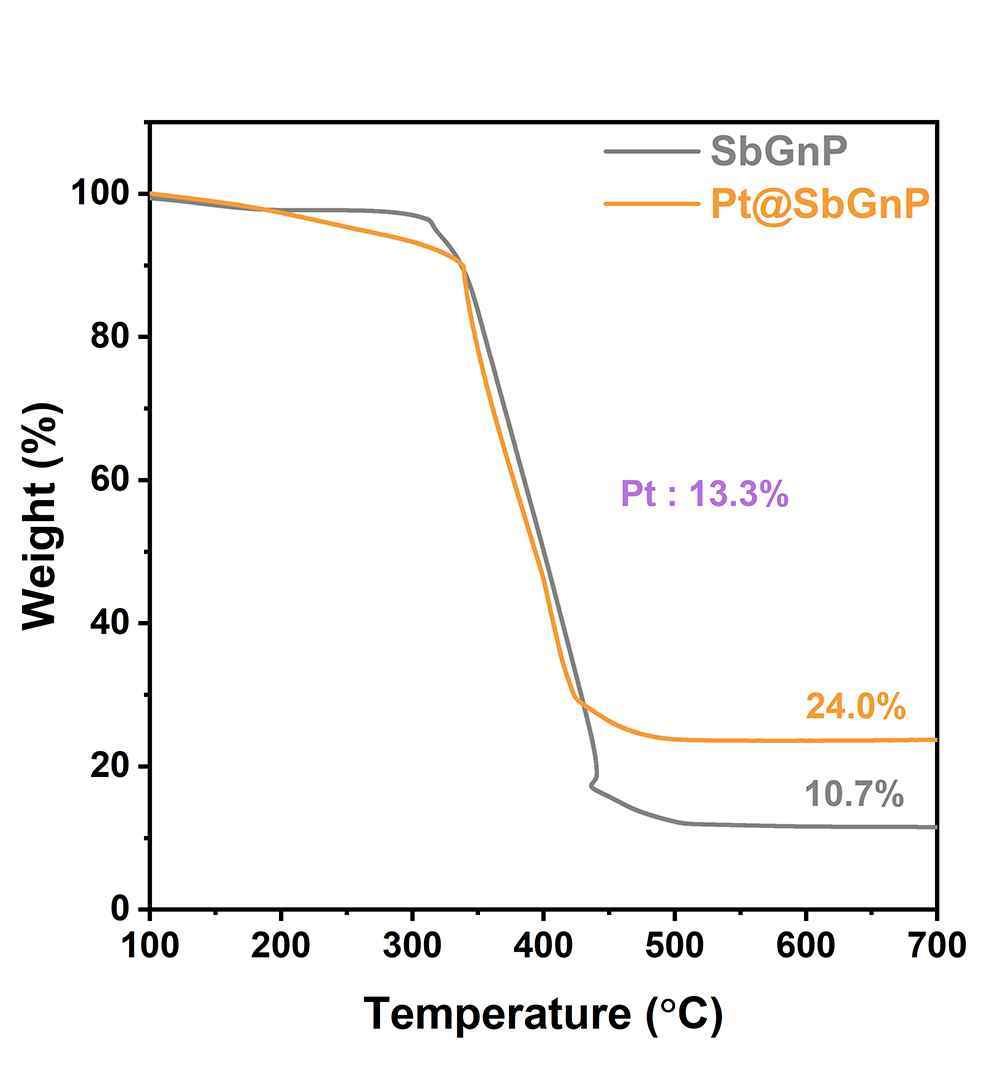


**Figure S3.** TGA thermograms of SbGnP and Pt@SbGnP obtained at a ramping rate of 10 °C min^−1^ under air atmosphere.


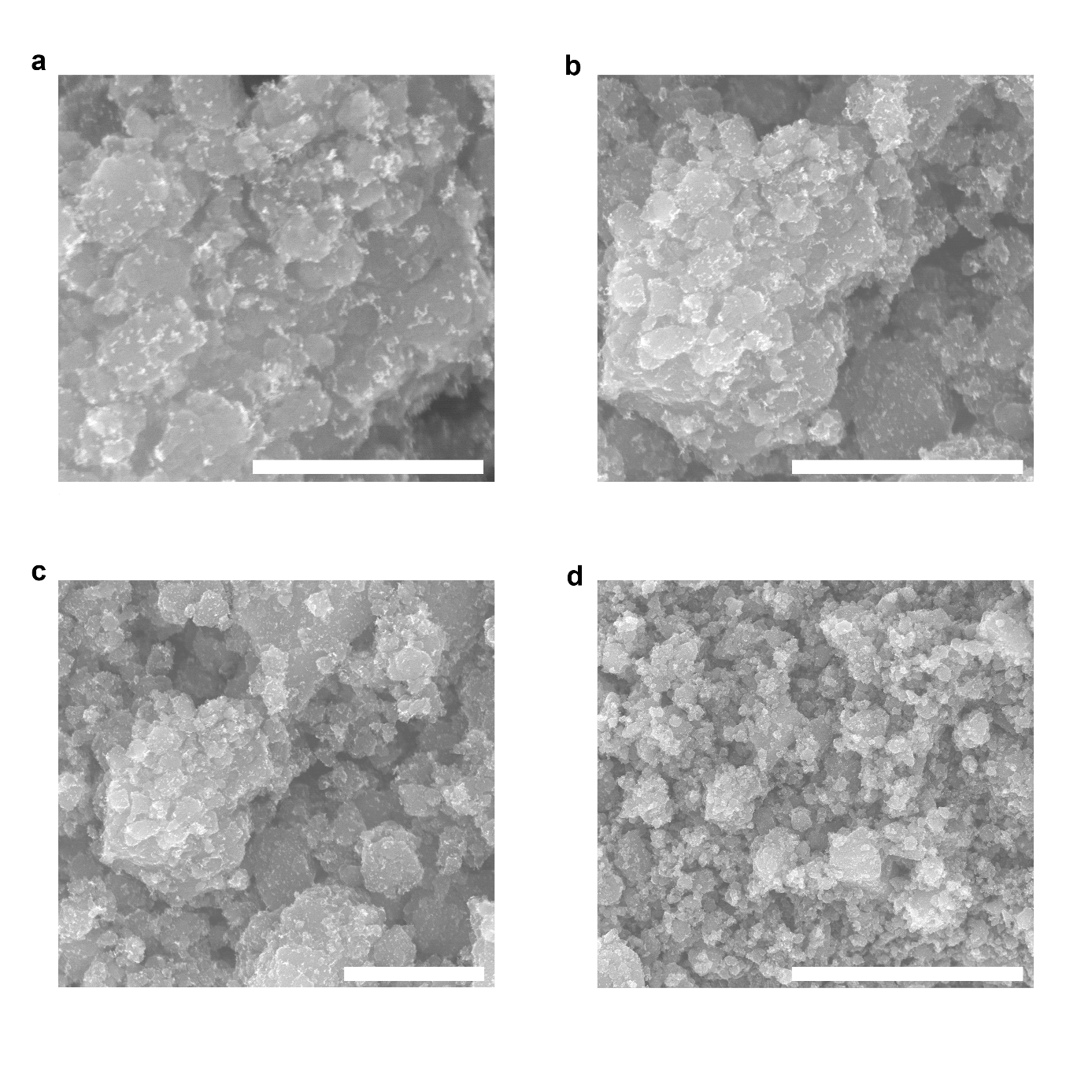


**Figure S4.** Scanning electron microscopy (SEM) images of Pt@SbGnP. SEM images of Pt@SbGnP at different magnifications.


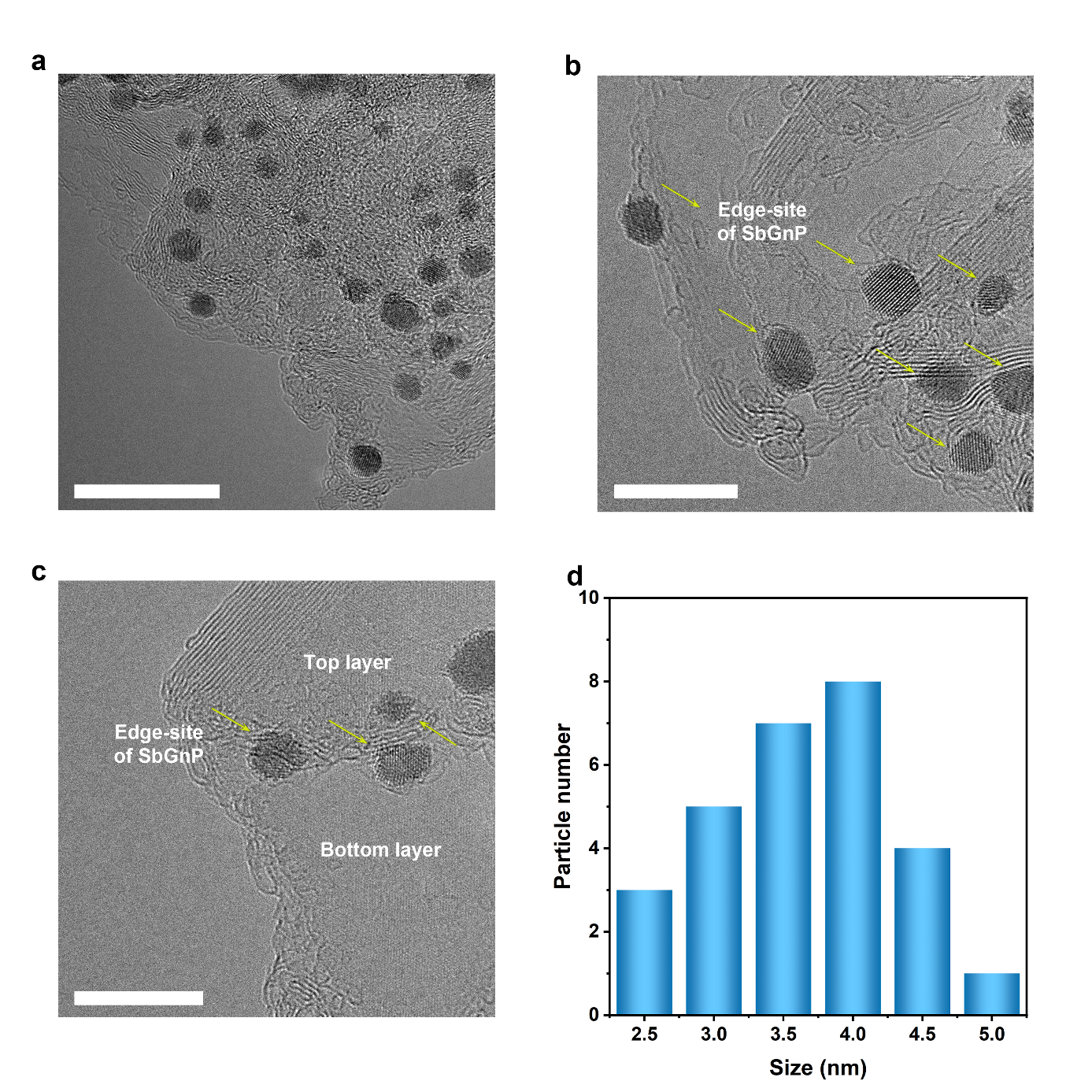


**Figure S5.** Transmission electron microscopy (TEM) images of the Pt@SbGnP. a) Normal TEM image. b-c) High resolution TEM image. d) The size distribution of Pt nanoparticles.


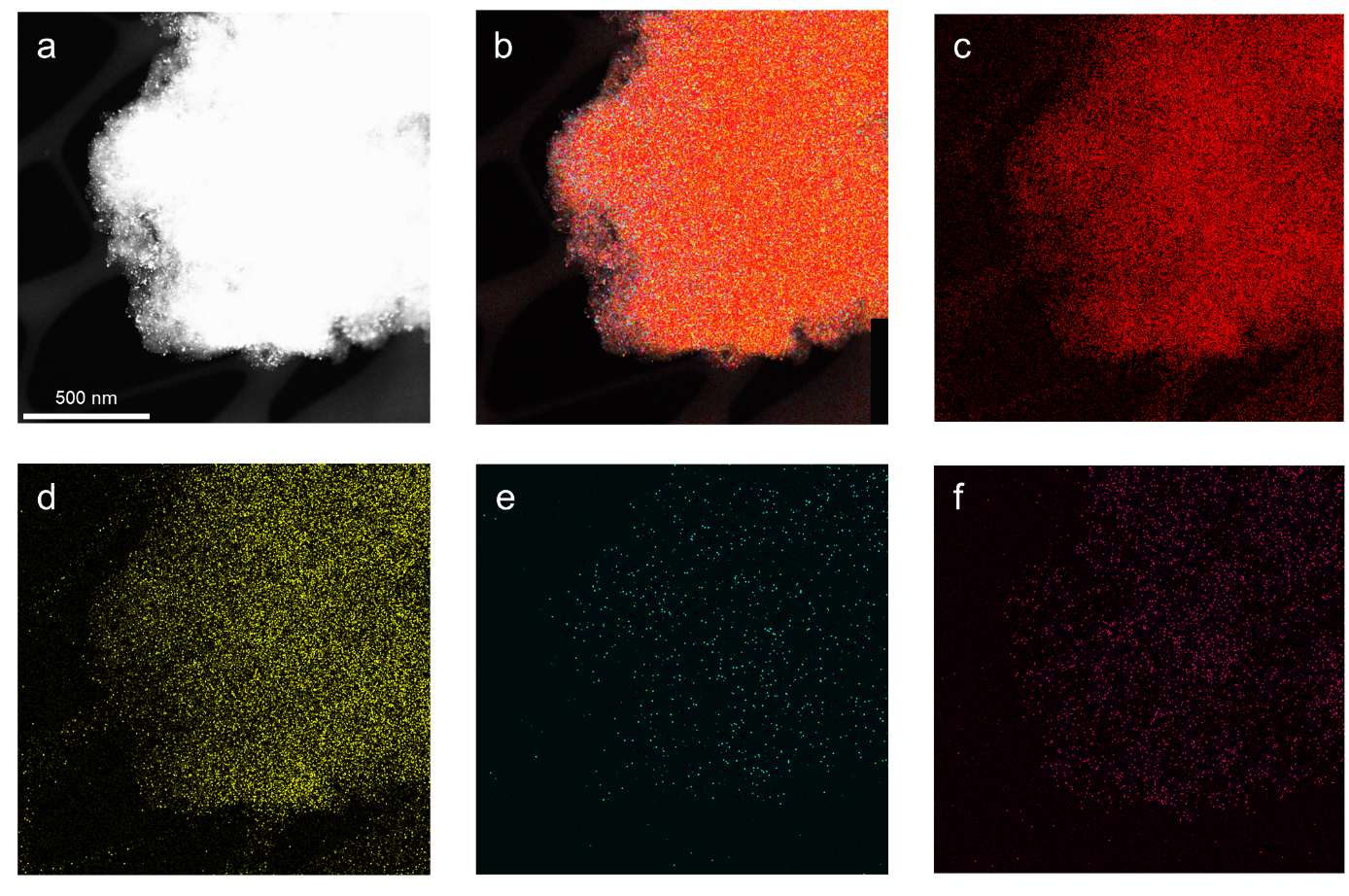


**Figure S6.** HAADF-STEM and EDS analyses of the Pt@SbGnP. a) High-angle annular dark-field scanning transmission electron microscopy (HAADF-STEM) image. Scanning transmission electron microscopy coupled energy-dispersive X-ray spectroscopy (STEM-EDS) element mappings: b) combined; c) carbon; d) oxygen; e) antimony; f) platinum.


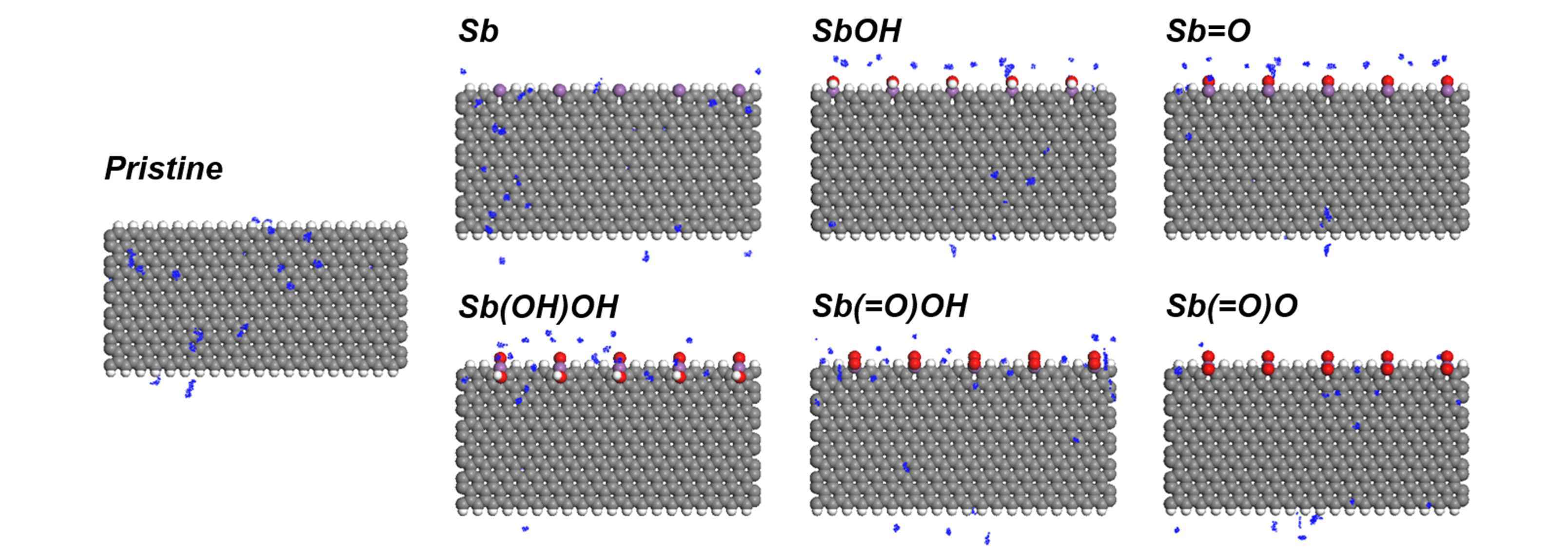


**Figure S7.** Density fields of PtCl_6_^2−^ ions adsorbed on the SbGnP models. Hydrogen, carbon, oxygen, and antimony atoms are colored with white, grey, red, and purple respectively. Blue dots represent favorable adsorption sites for Pt precursors, when five pairs of H_2_PtCl_6_ ions were adsorbed.


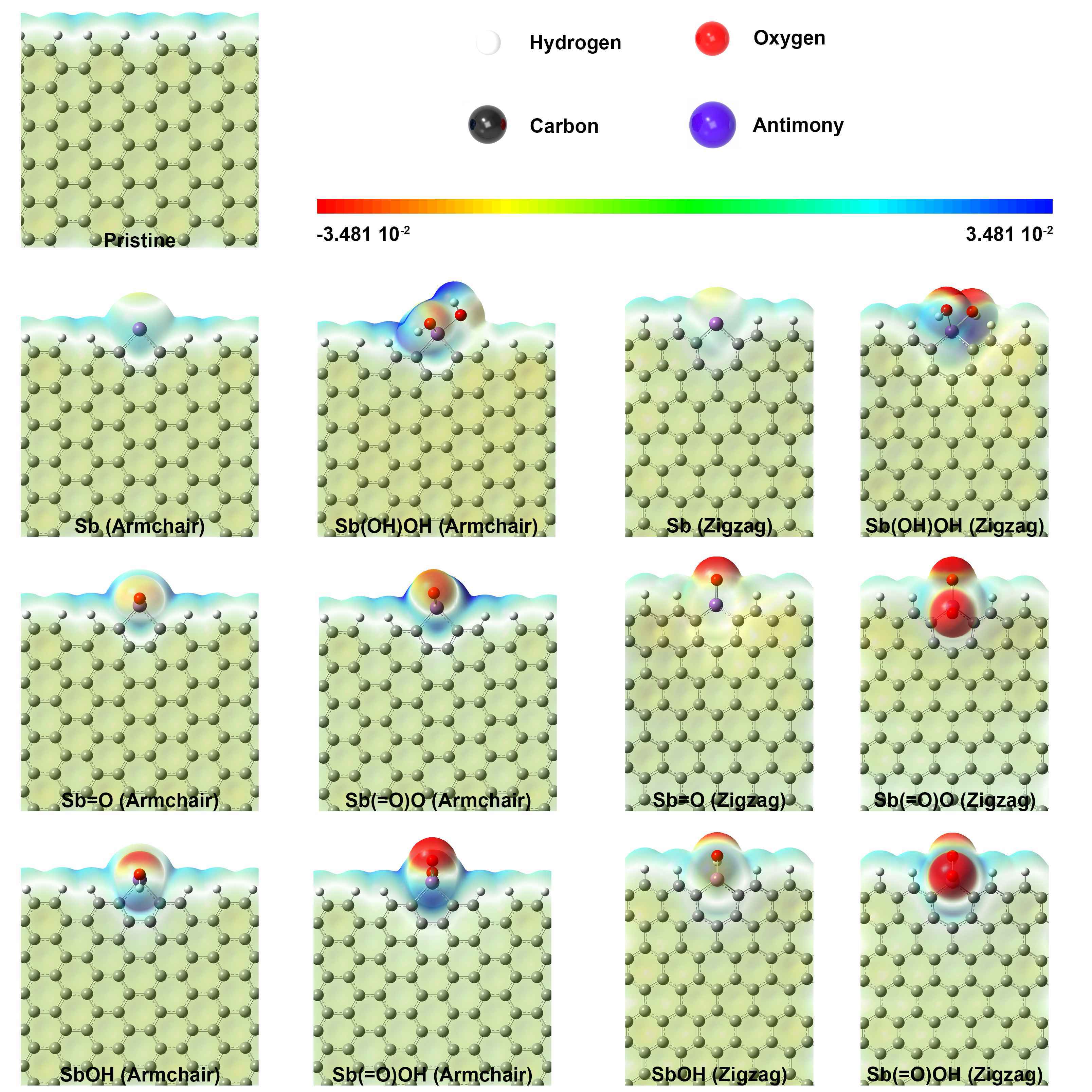


**Figure S8.** Electron density maps of SbGnP models. Hydrogen, carbon, oxygen, and antimony atoms are colored with white, black, red, and purple respectively.


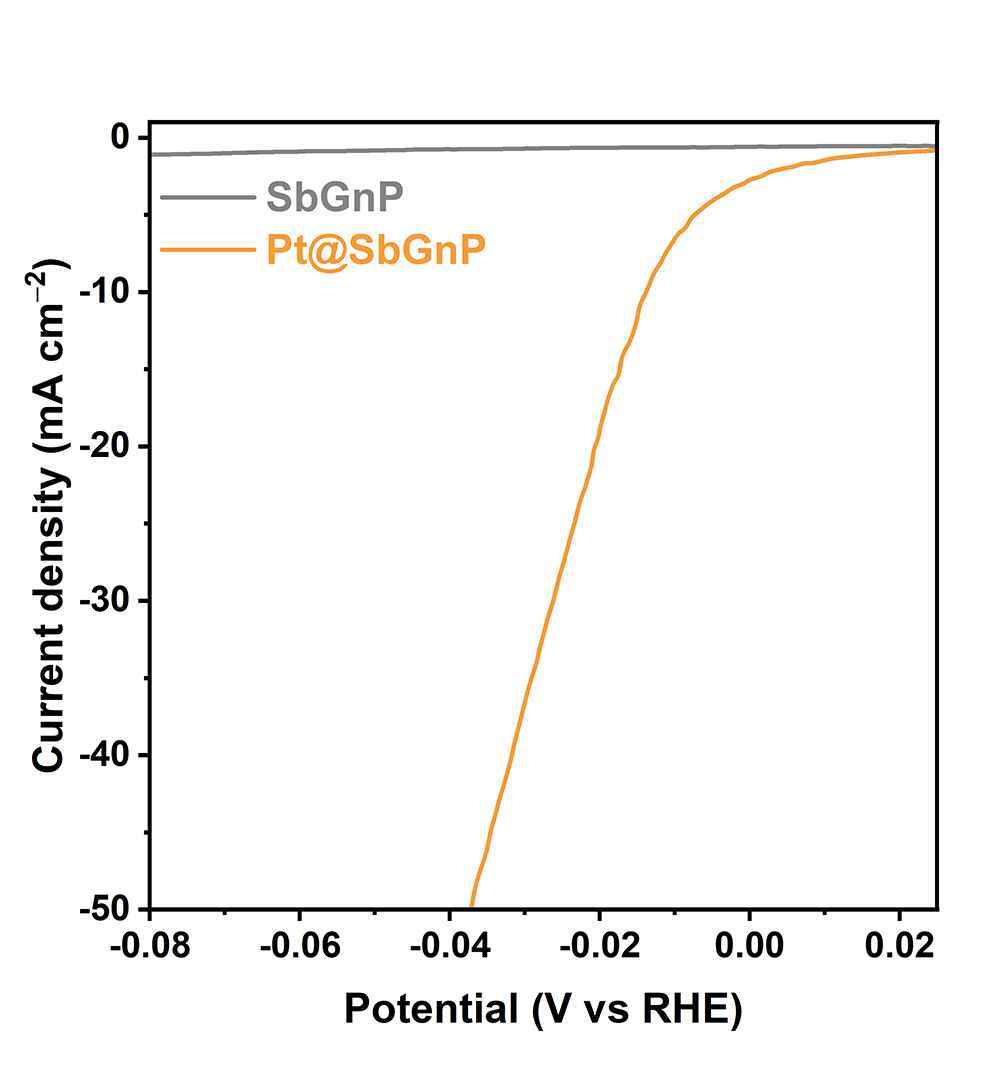


**Figure S9.** Linear sweep voltammetry (LSV) curves of SbGnP and Pt@SbGnP. Polarization curves of SbGnP and the Pt@SbGnP catalysts at a scan rate of 5 mV s^−1^ in 0.5 ᴍ aq. H_2_SO_4_ solution.


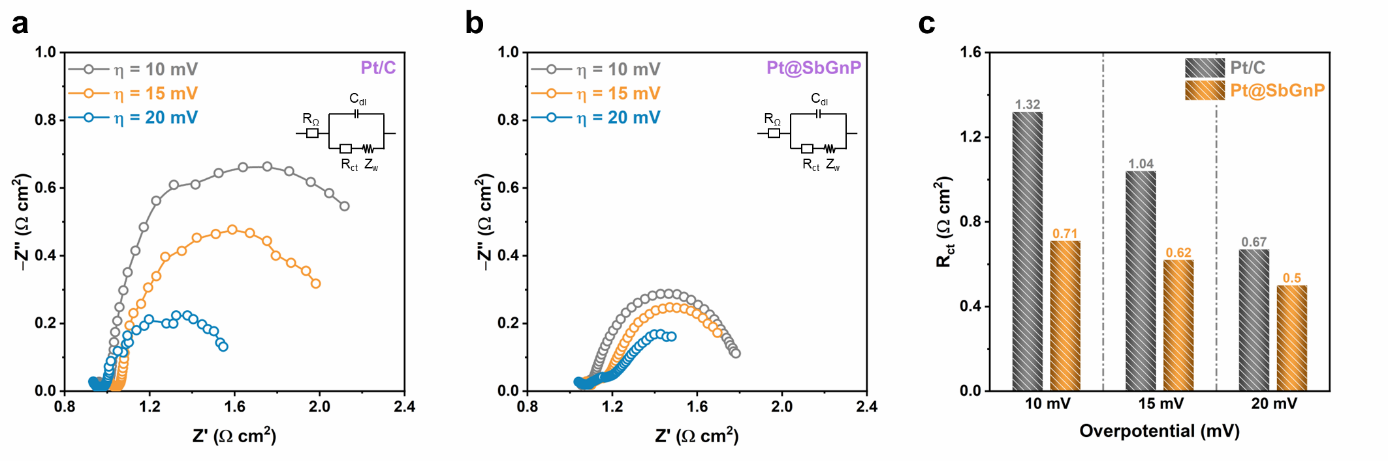


**Figure S10.** Nyquist plots of Pt/C and Pt@SbGnP. Comparison of electrochemical impedance spectroscopy (EIS) curves at specific overpotentials (10, 15 and 20 mV) in 0.5 ᴍ aq. H_2_SO_4_ solution: a) Pt/C; b) Pt@SbGnP. c) Charge transfer resistances (R_ct_) calculated from EIS curves.


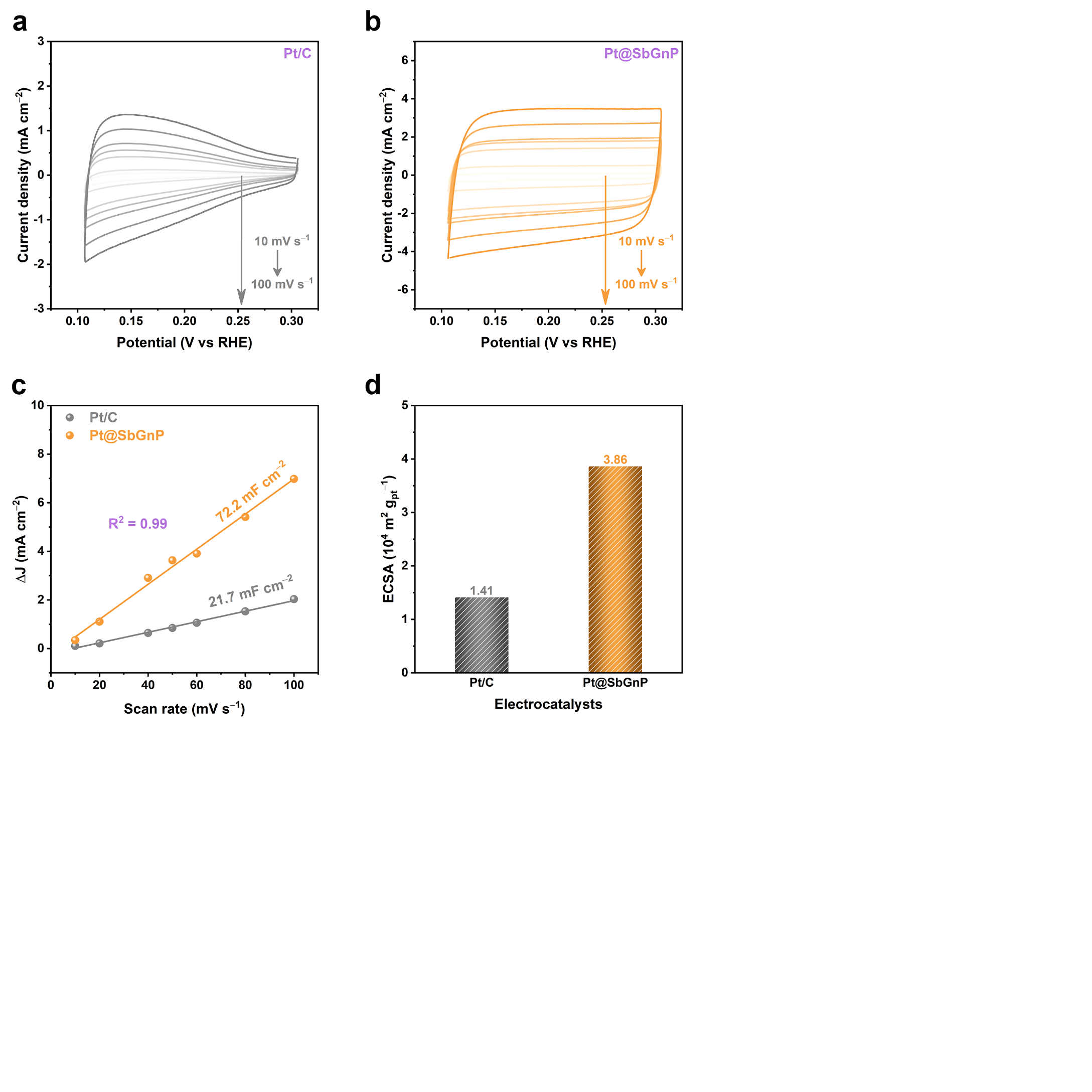


**Figure S11.** Cyclic voltammetry (CV) analysis of Pt/C and Pt@SbGnP. Cyclic voltammetry curves at scan rates from 10 to 100 mV s^−1^: a) Pt/C; b) Pt@SbGnP. c) Scan rate dependence of the current densities of Pt/C and Pt@SbGnP. d) Calculated ECSA values of Pt/C and Pt@SbGnP.


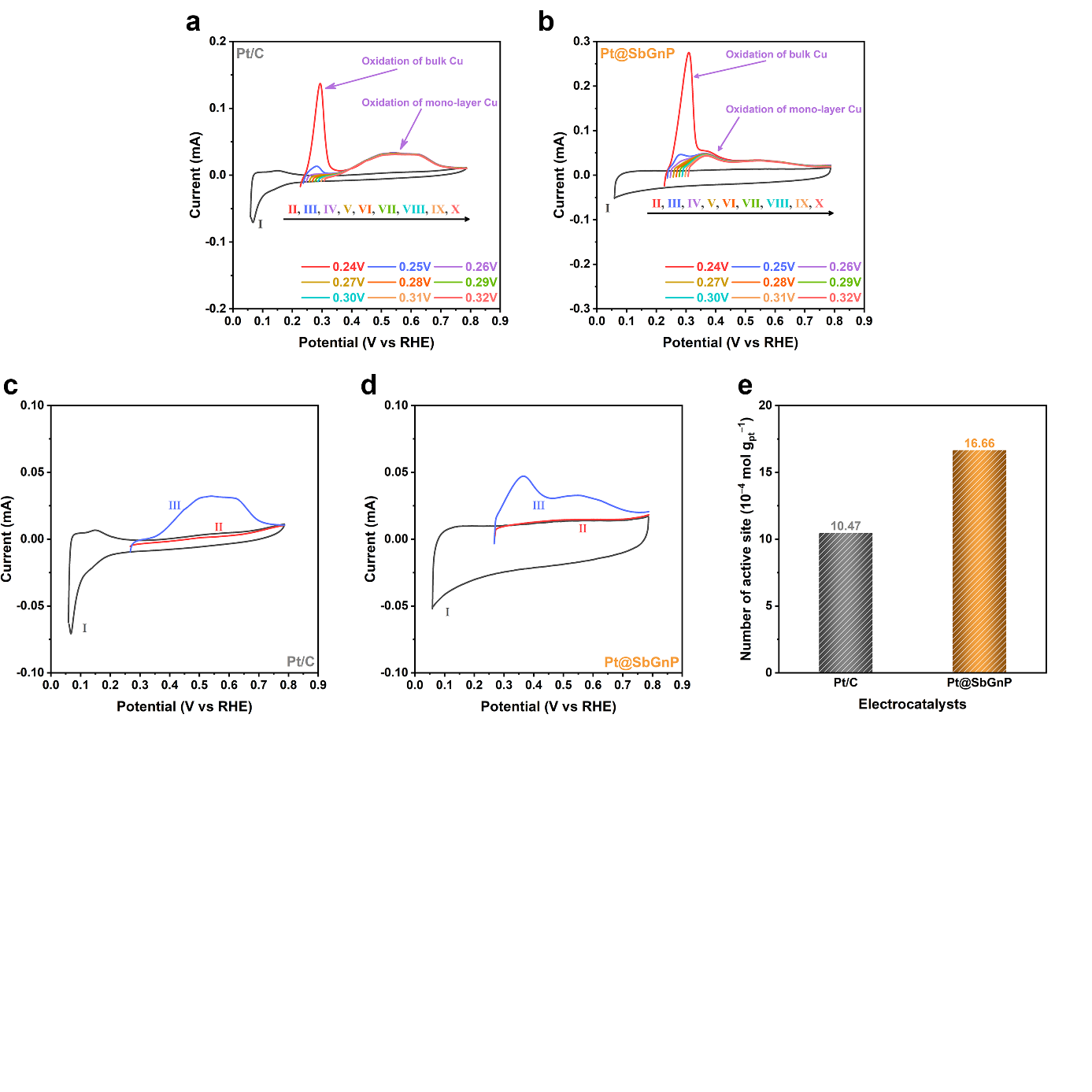


**Figure S12.** Copper under-potential deposition (UPD) analysis. Copper UPD curves in 0.5 ᴍ aq. H_2_SO_4_ solution with/without 5 mM CuSO_4_: a) Pt/C; b) Pt@SbGnP. The electrodes were polarized at 0.24 V for 100 s to form the UPD layer. Copper UPD in 0.5 ᴍ aq. H_2_SO_4_ solution with/without 5 mM CuSO_4_: c) Pt/C; d) Pt@SbGnP. The electrodes were polarized at 0.28 V for 100 s to form the UPD layer. e) Calculated number of active sites.


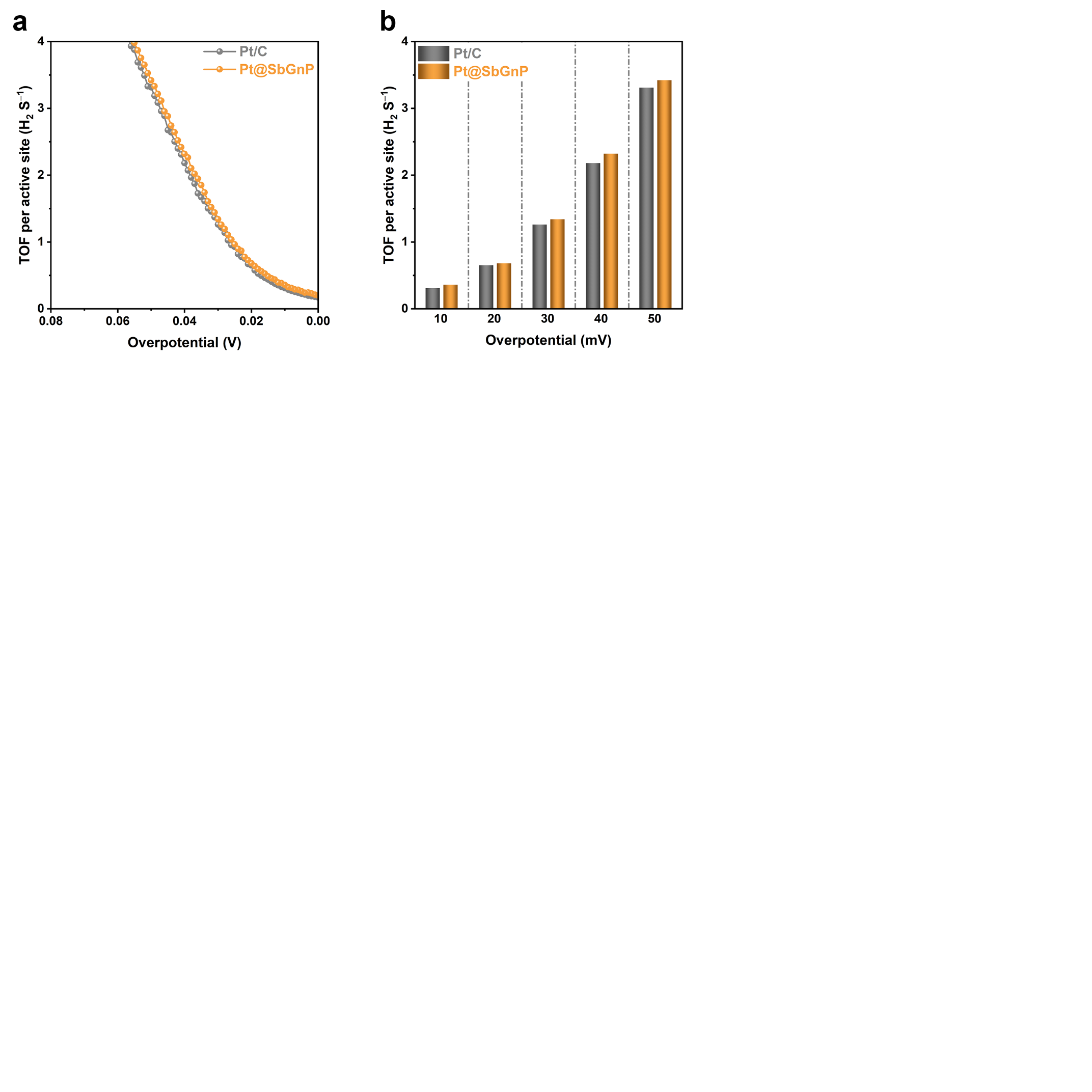


**Figure S13.** Turnover Frequency of Pt/C and Pt@SbGnP. a) Plot of TOF as a function potential; b) TOF value at specific overpotentials (10, 20, 30, 40 and 50 mV).


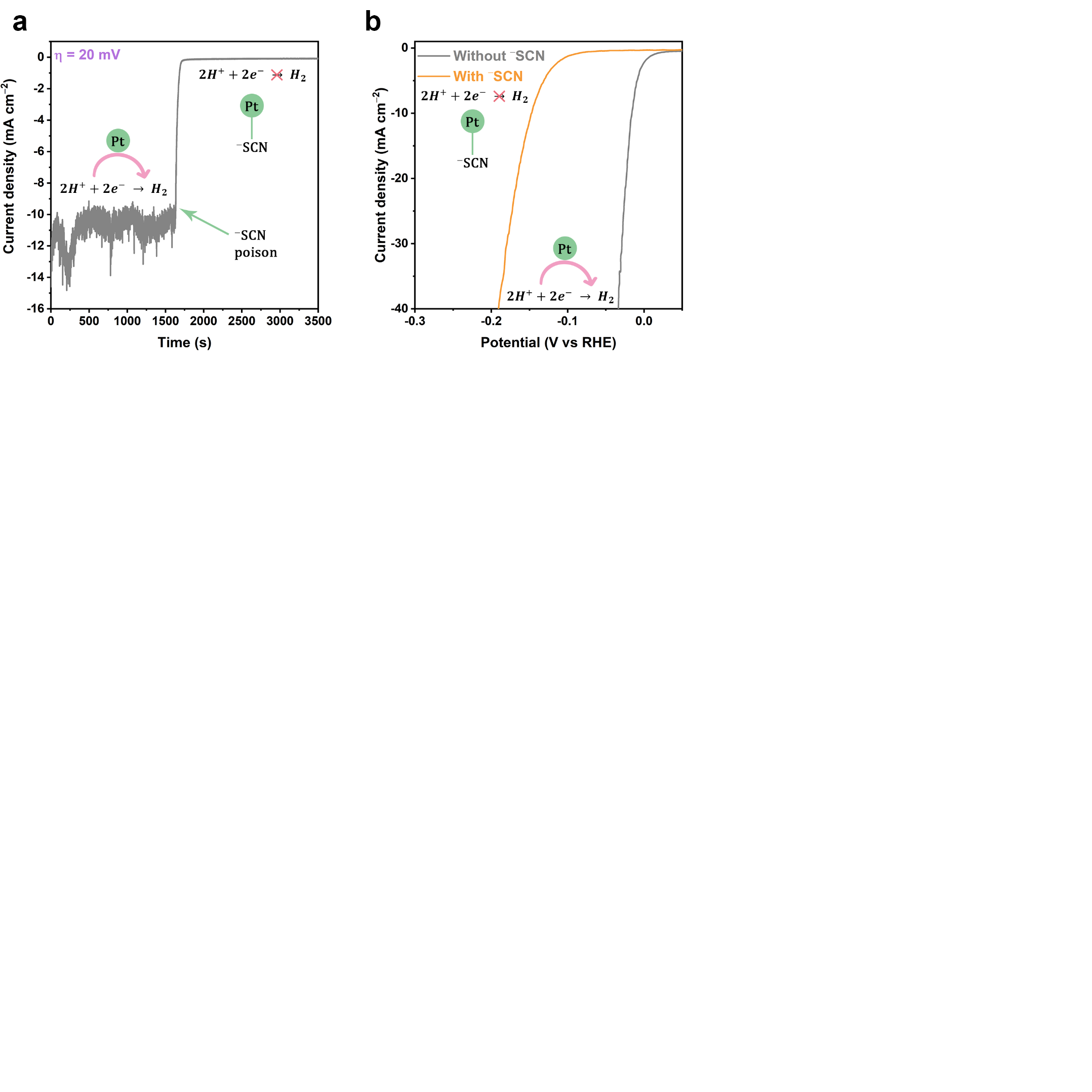


**Figure S14.** Thiocyanate ion (^−^SCN) poisoning test. a) Chronoampherometry (CA) curves of Pt@SbGnP before and after the addition of ^−^SCN ions to the 0.5 ᴍ aq. H_2_SO_4_ solution. b) Linear sweep voltammetry (LSV) curves of Pt@SbGnP before and after adding ^−^SCN ions to the 0.5 ᴍ aq. H_2_SO_4_ solution. The insets in a-b) illustrate the HER pathway with/without ^–^SCN.


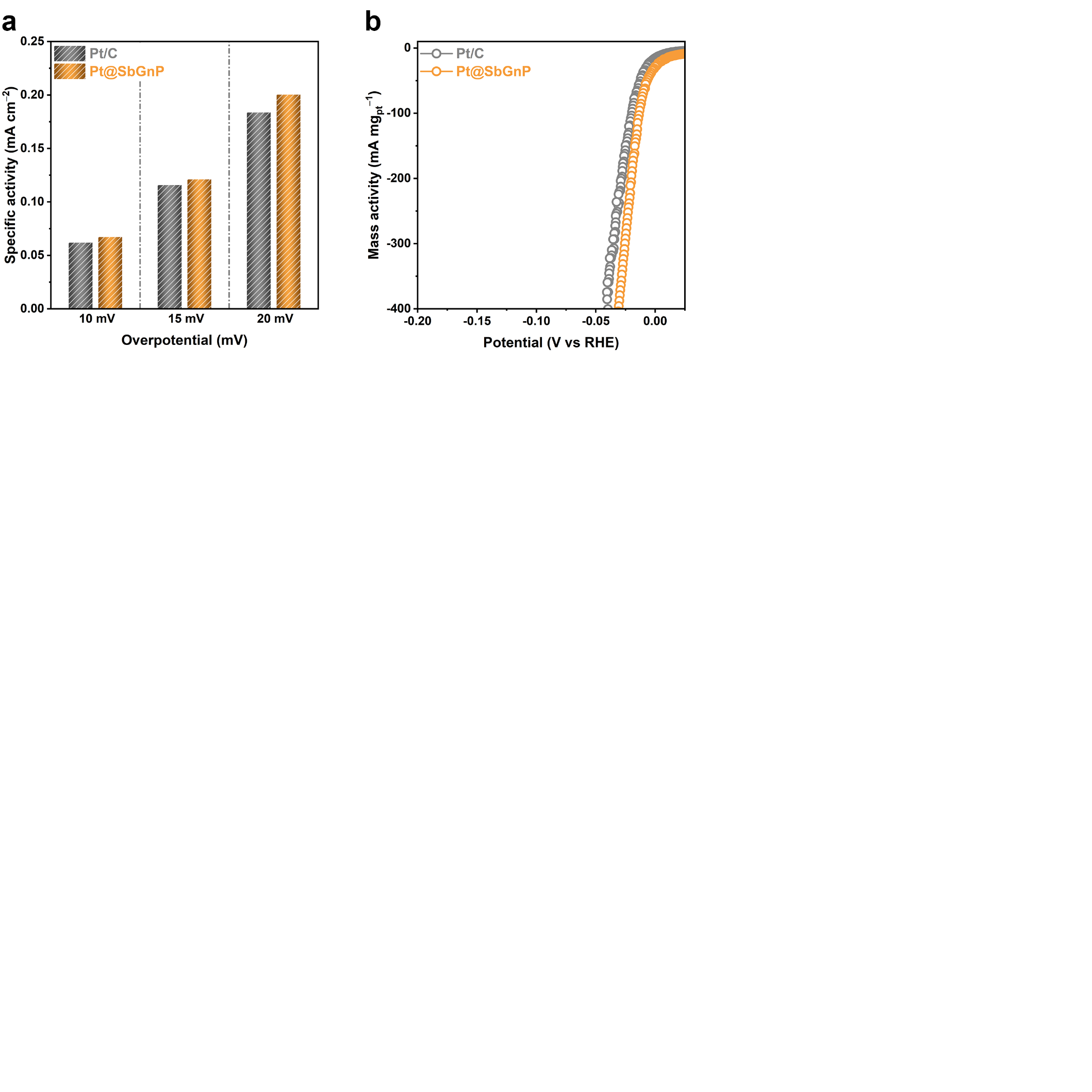


**Figure S15.** Specific activity and mass activity. a) Specific activities at different overpotentials (10, 15, and 20 mV). b) Mass activities in Ar-saturated 0.5 ᴍ aq. H_2_SO_4_ solution.


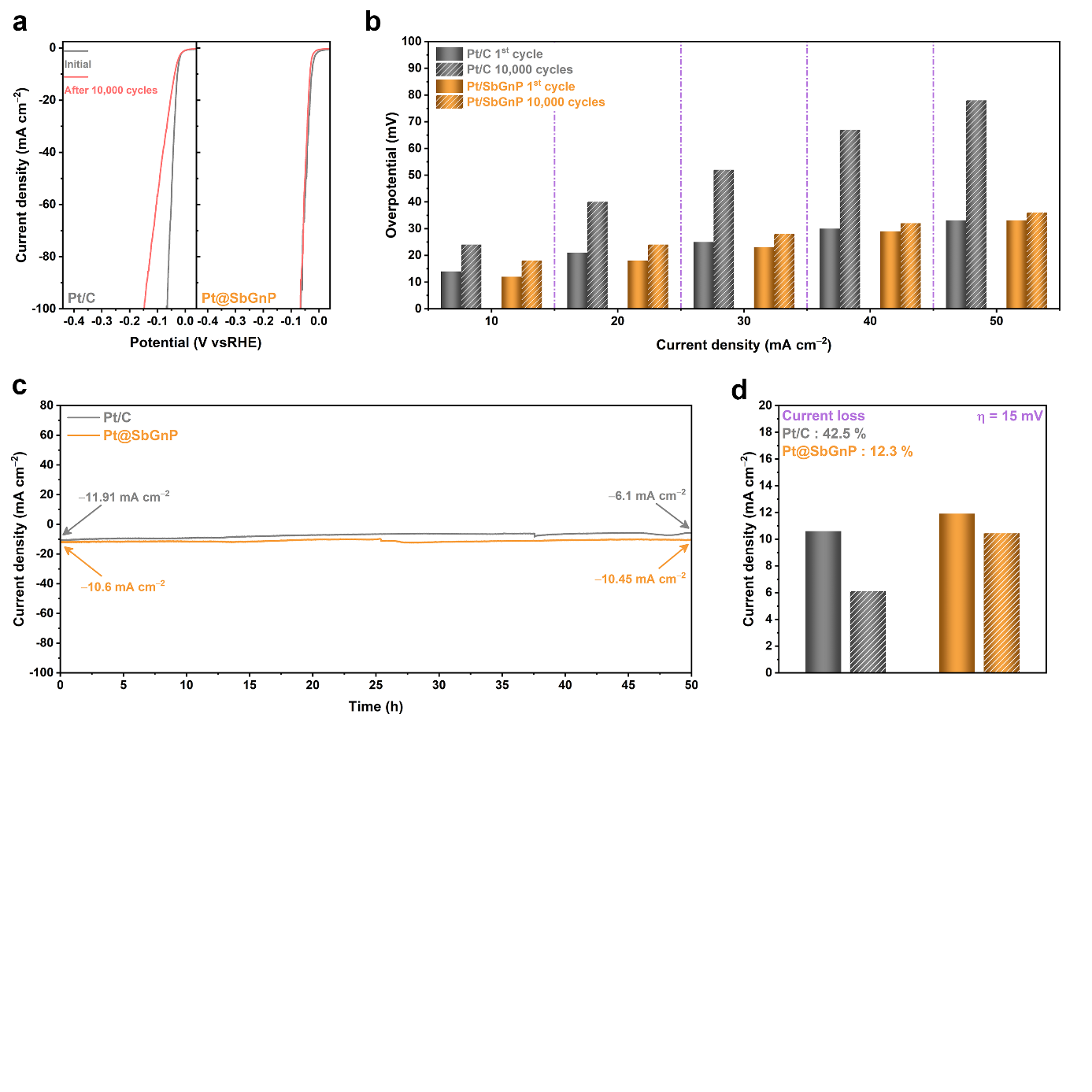


**Figure S16.** LSV curves before/after CV test and time-dependent current density curve. a) The polarization curves before and after 10,000 CV potential cycles. b) Comparison of overpotential changes at different current densities after 10,000 CV potential cycles. c) Current-time (I vs. t) stability curves up to 50 h duration were recorded in 0.5 ᴍ aq. H_2_SO_4_ solutions. The slight current loss can be attributed to the peeling off the catalysts during the release of H_2_ bubbles. d) The losses of current densities before and after 50 h.


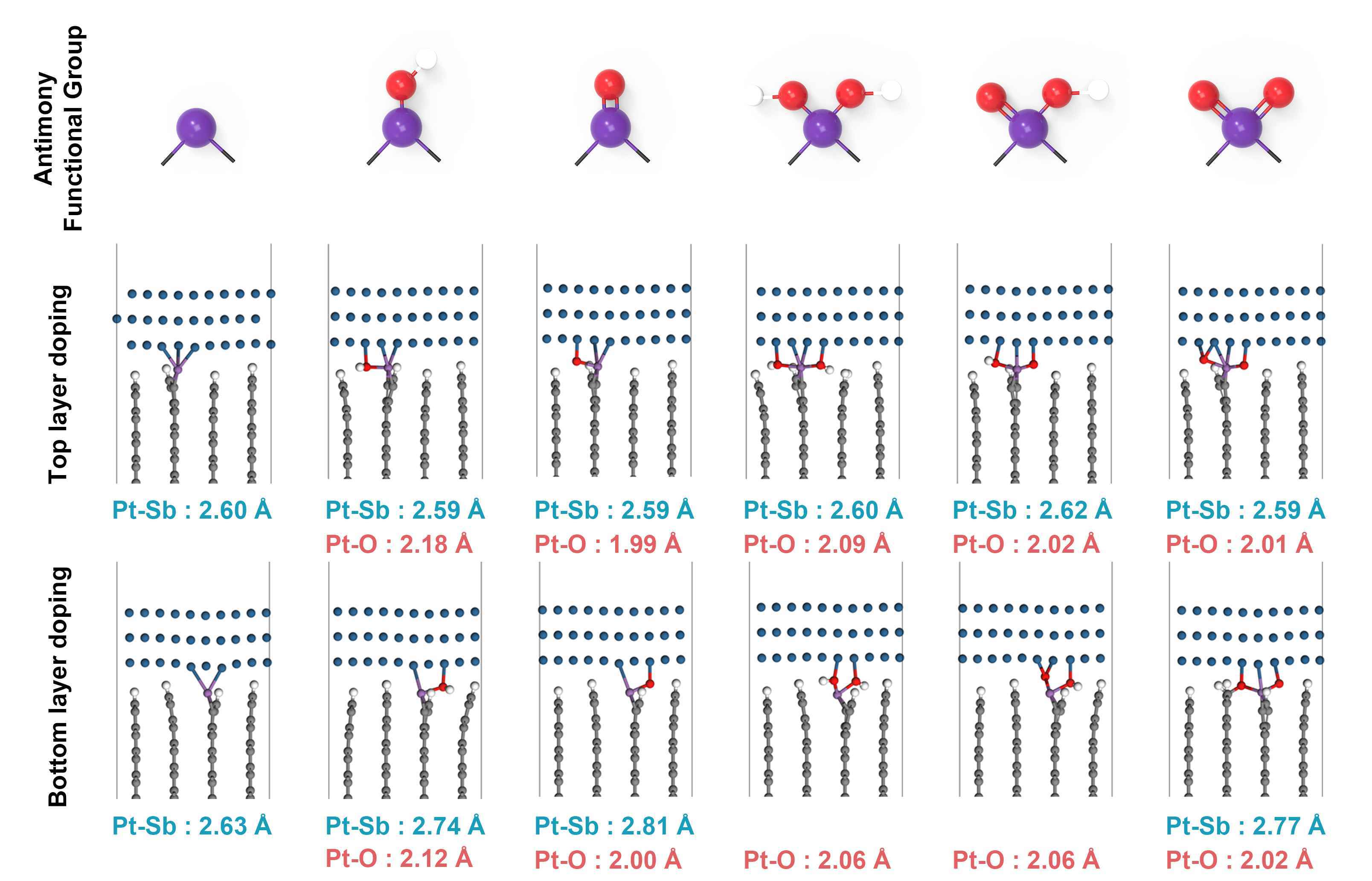


**Figure S17.** Bonding configurations formed between the Pt slab and the SbGnP layer. The shortest Pt-Sb and Pt-O bonds are noted under each image. Note that the Pt-Sb distances were not close enough to form bonds with the Sb(OH)OH and Sb(=O)OH groups in the bottom layer doping. The platinum, hydrogen, carbon, oxygen, and antimony atoms are colored by dark blue, white, grey, red, and purple, respectively.


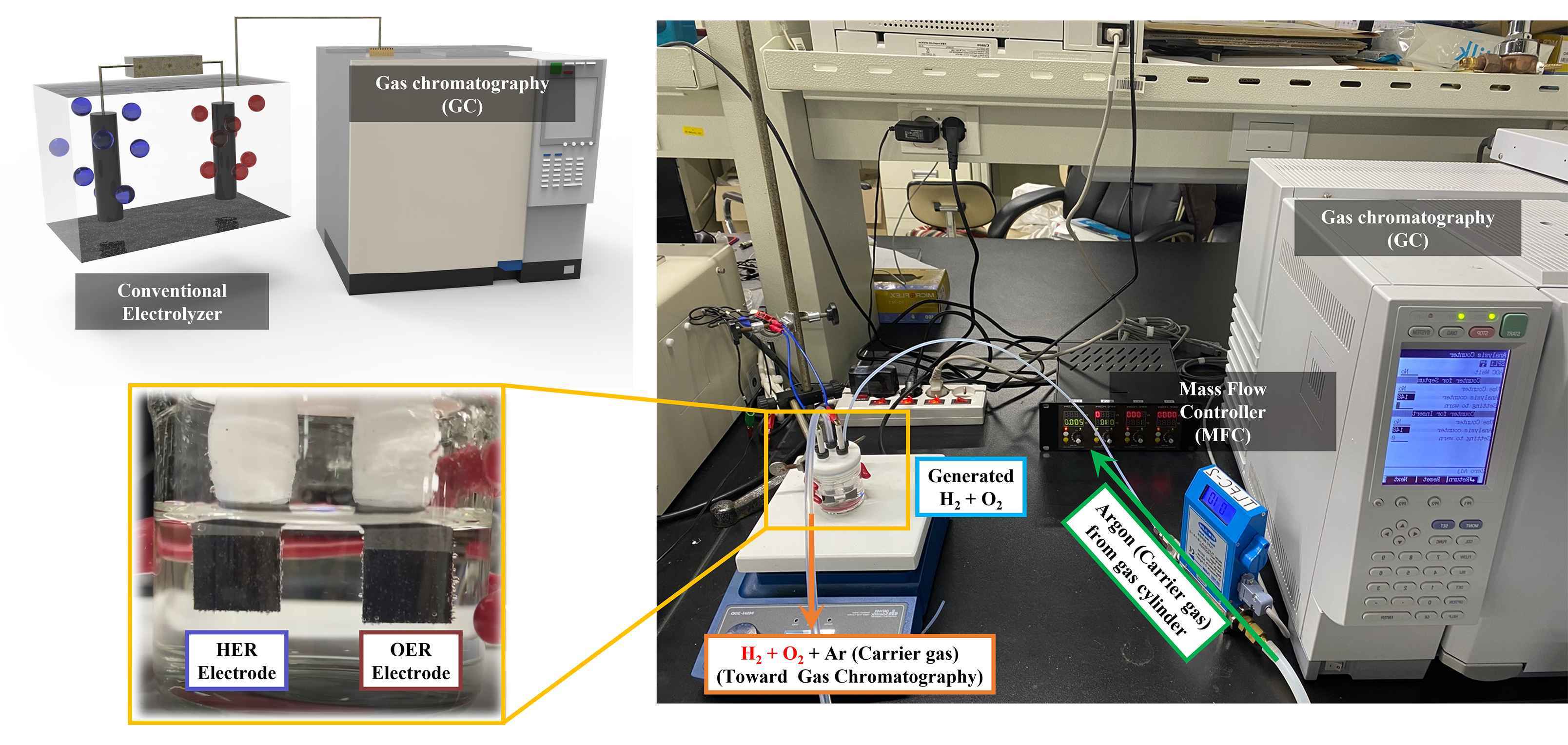


**Figure S18.** System configuration for evaluating hydrogen quantitative. Schematic diagram of water electrolysis setup for the quantitative measurement of hydrogen production.


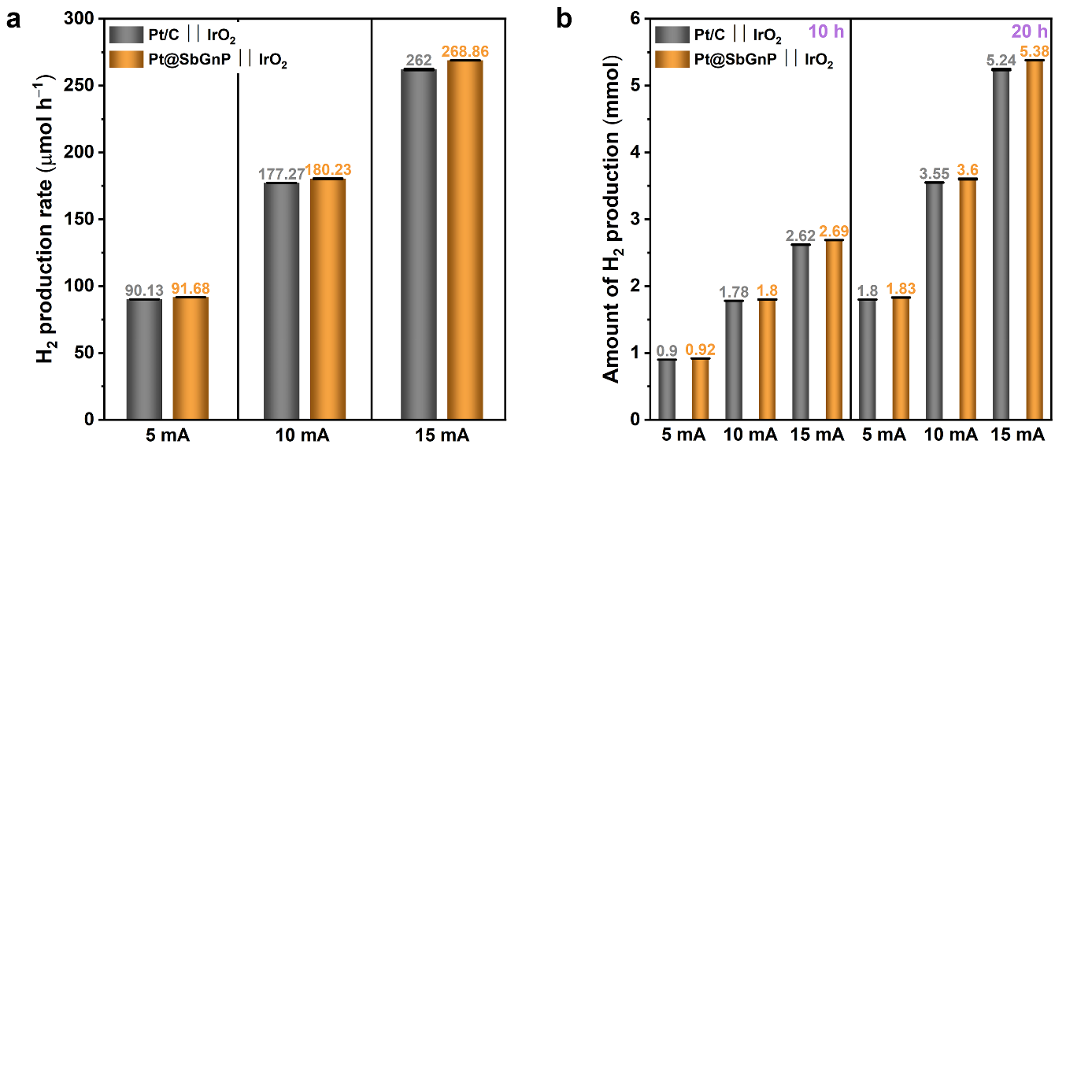


**Figure S19.** Hydrogen production rate and amount of hydrogen production. a) Average hydrogen production rate and b) amount of hydrogen production at specific currents of 5, 10 and 15 mA. The error bar reflects the results of three device measurements.


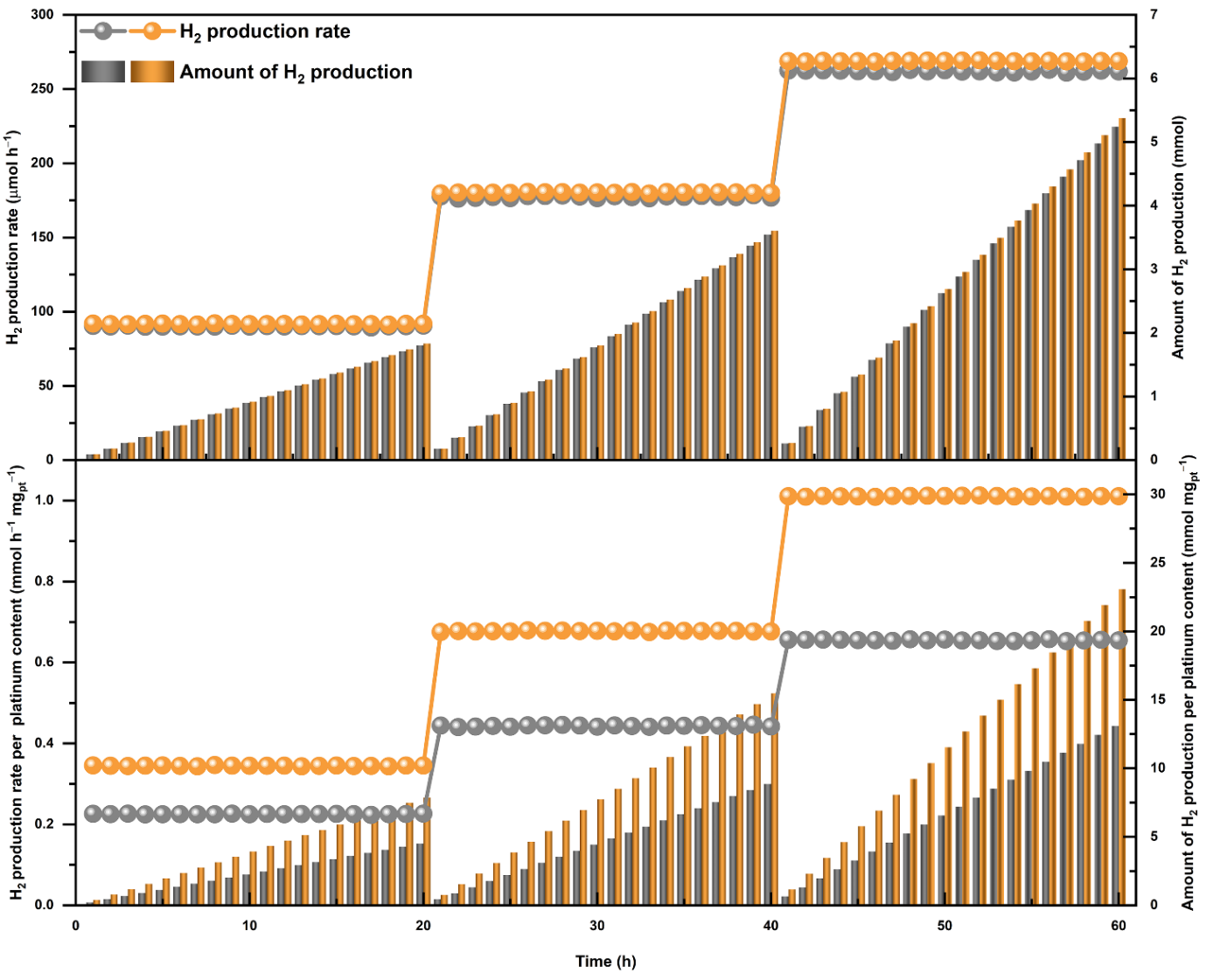


**Figure S20.** Hydrogen production rate and hydrogen production volume compared to platinum content. The hydrogen production rate and hydrogen production rate per platinum content at specific currents of 5, 10 and 15 mA.


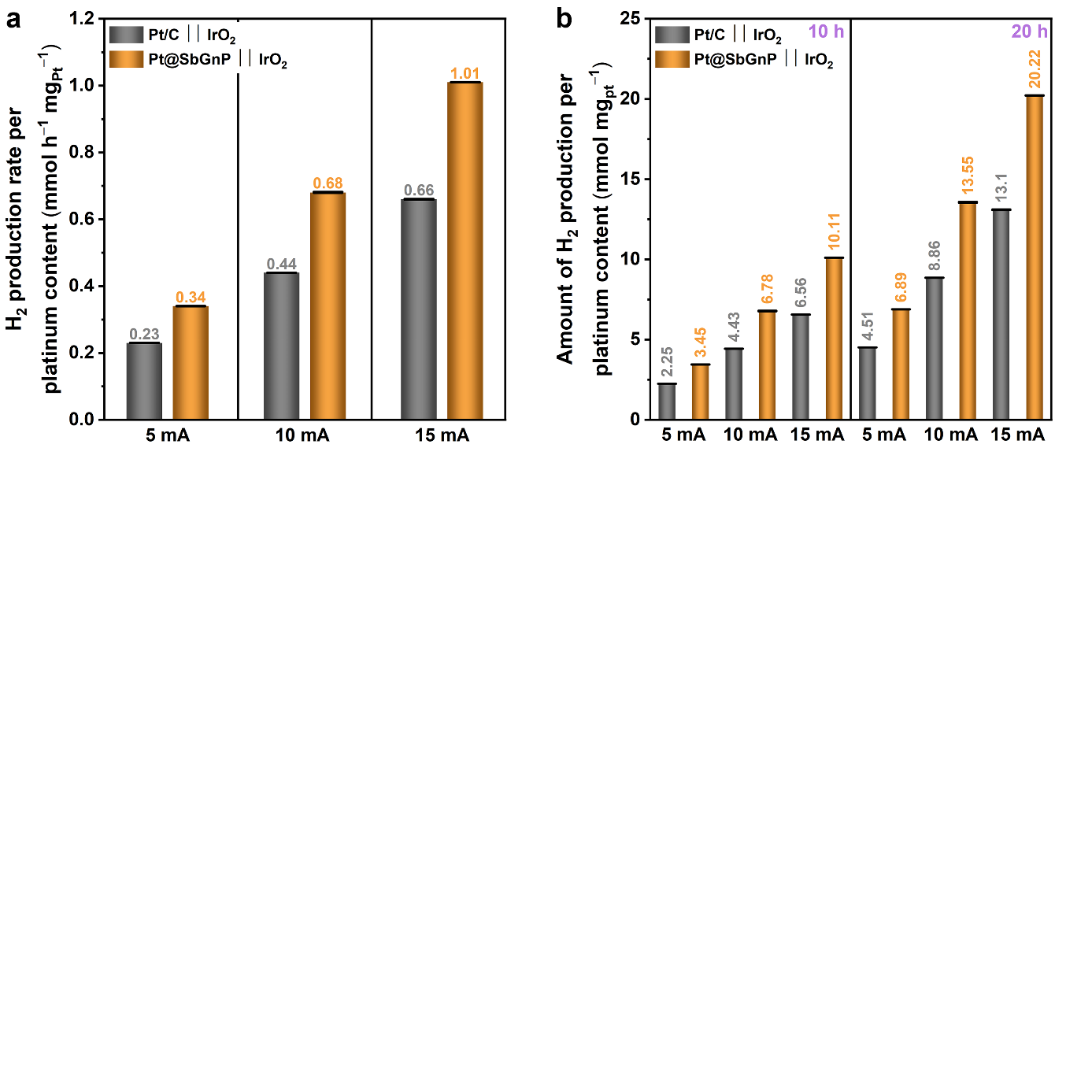


**Figure S21.** Hydrogen production rate per platinum content and amount of hydrogen production per platinum content. a) Average hydrogen production rate per platinum content and b) amount of hydrogen production per platinum content at specific currents of 5, 10 and 15 mA. The error bar reflects the results of three device measurements.


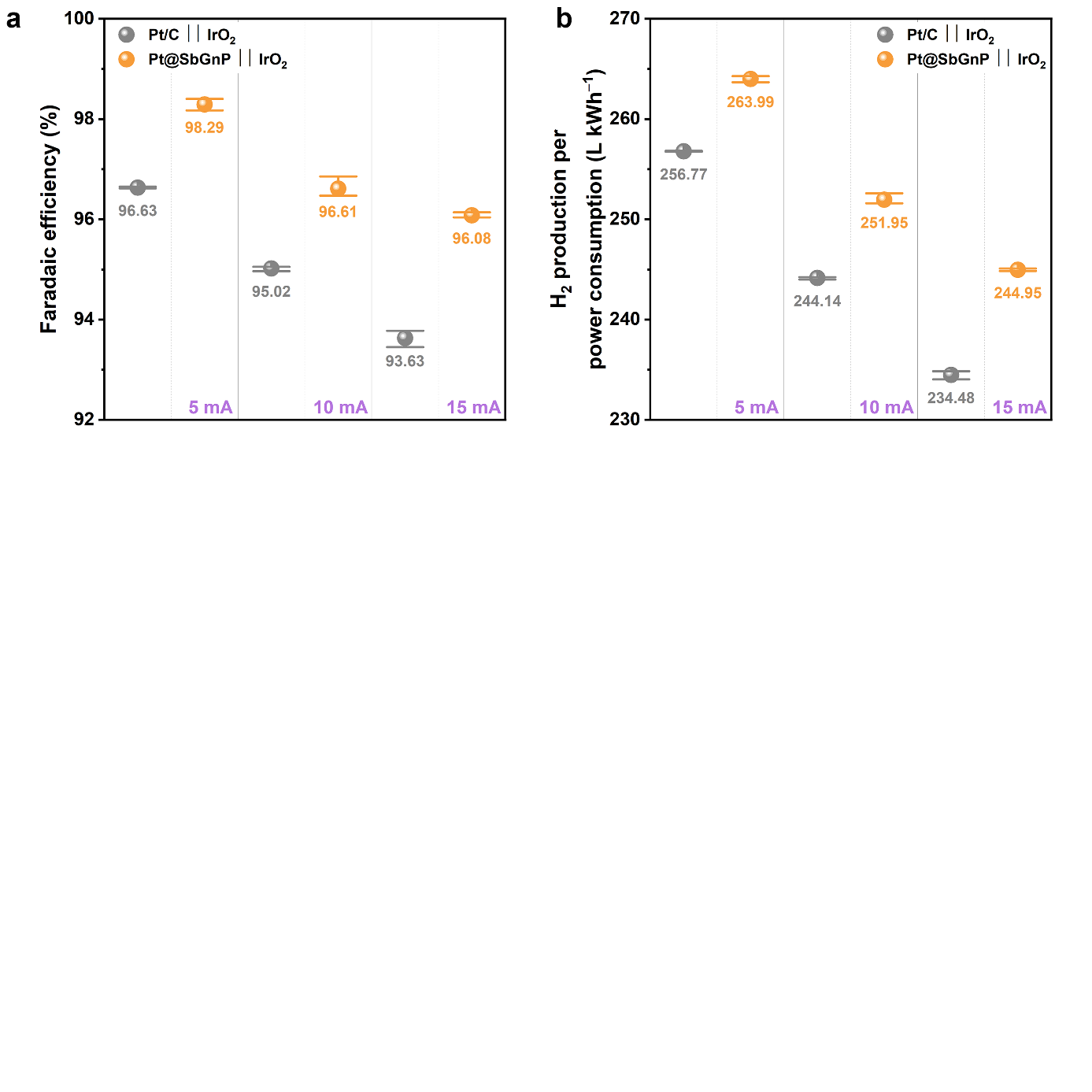


**Figure S22.** Faradaic efficiency and hydrogen production per power consumption. a) Average faradaic efficiency and b) hydrogen production per power consumption at specific currents of 5, 10 and 15 mA. The error bar reflects the results of three device measurements.


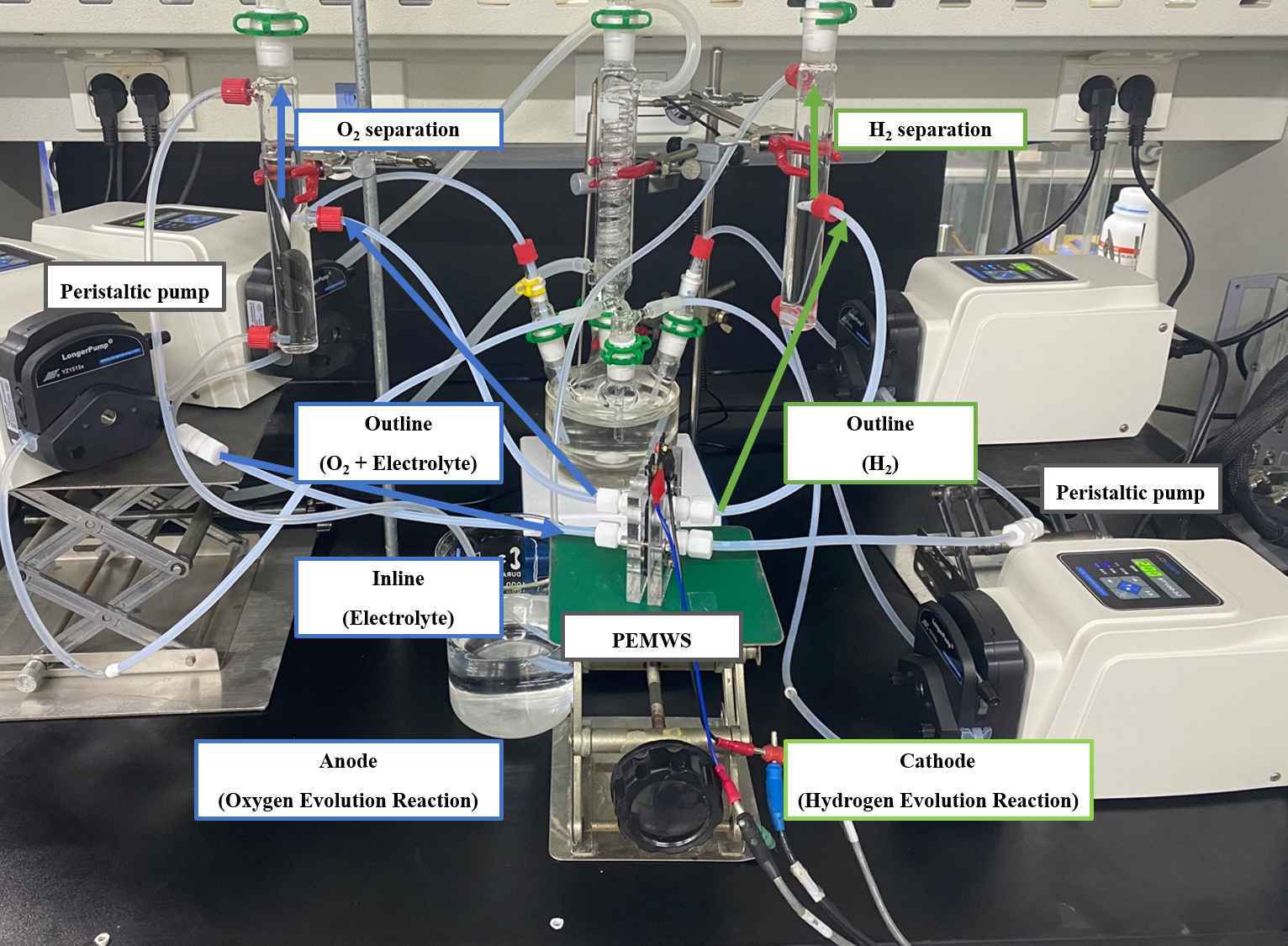


**Figure S23.** Digital photograph of home-made PEMWE measurement system.


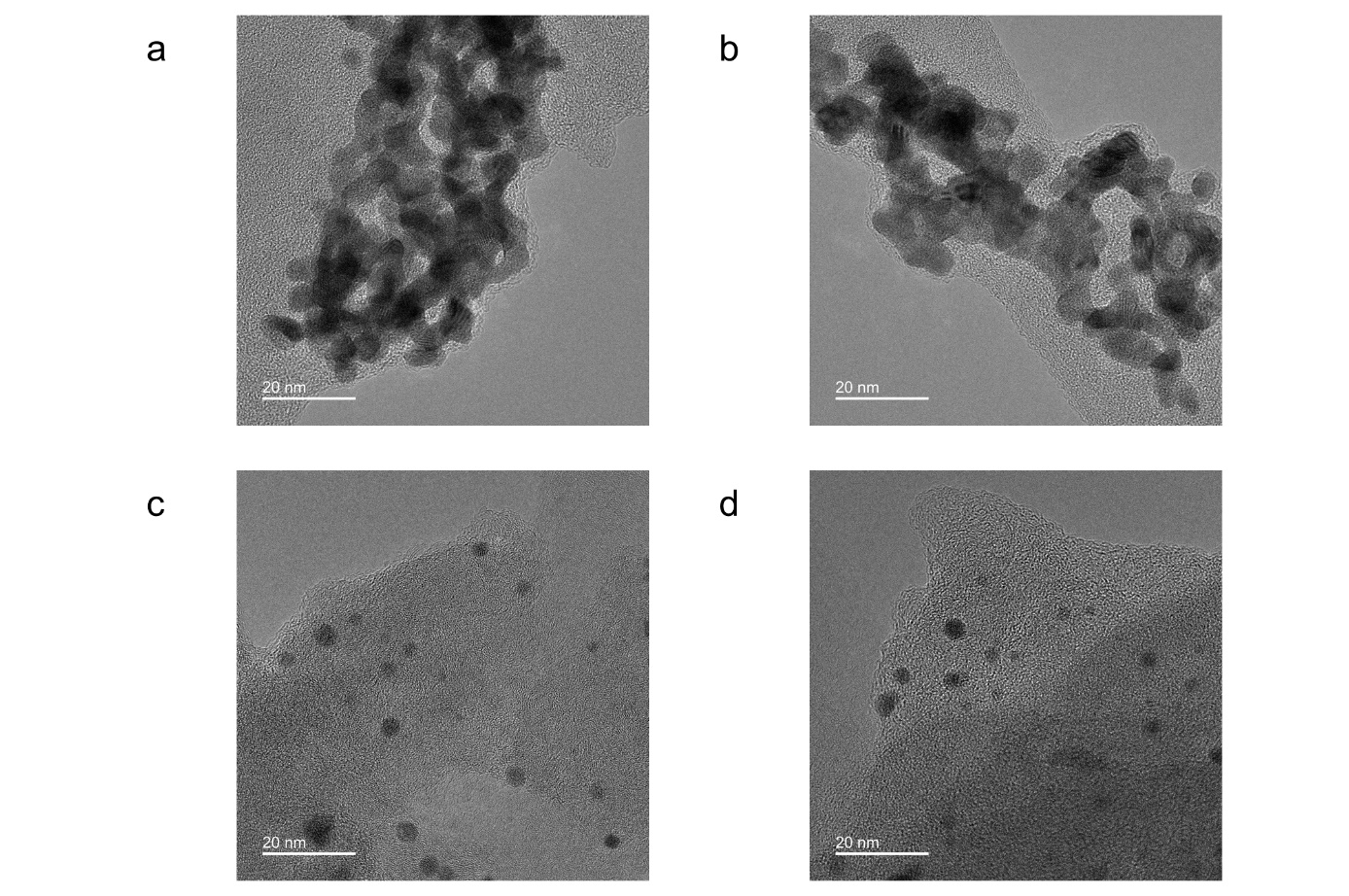
**Figure S24.** HR-TEM images after the durability test: a-b) Pt/C at low and high magnifications, respectively; c-d) Pt@SbGnP at low and high magnifications, respectively.


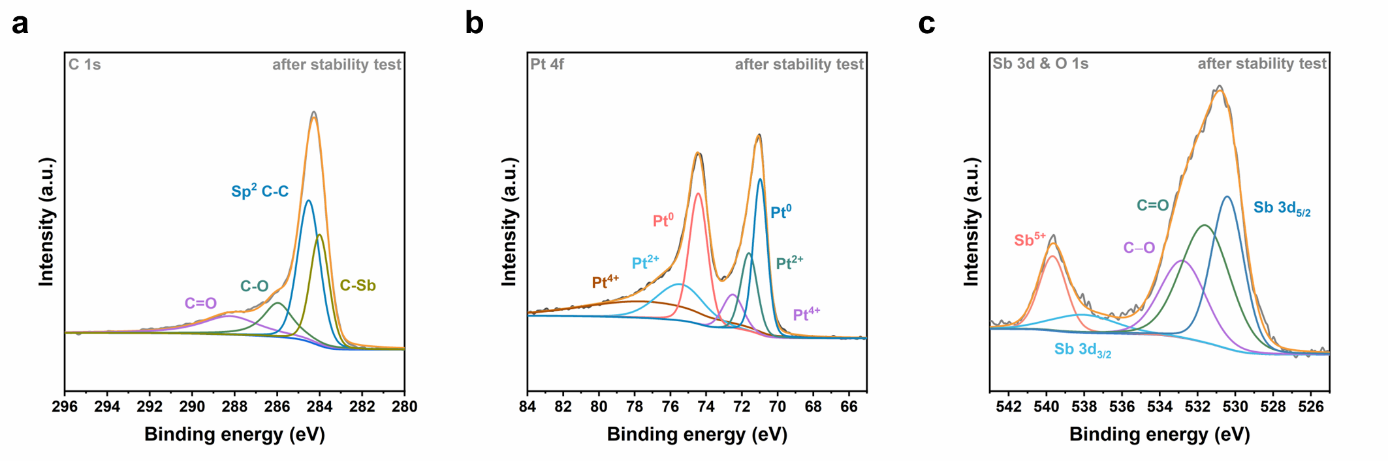


**Figure R25.** High-resolution spectra after the durability test: a) C 1s; b) Pt 4f; c) Sb 3d & O 1s.


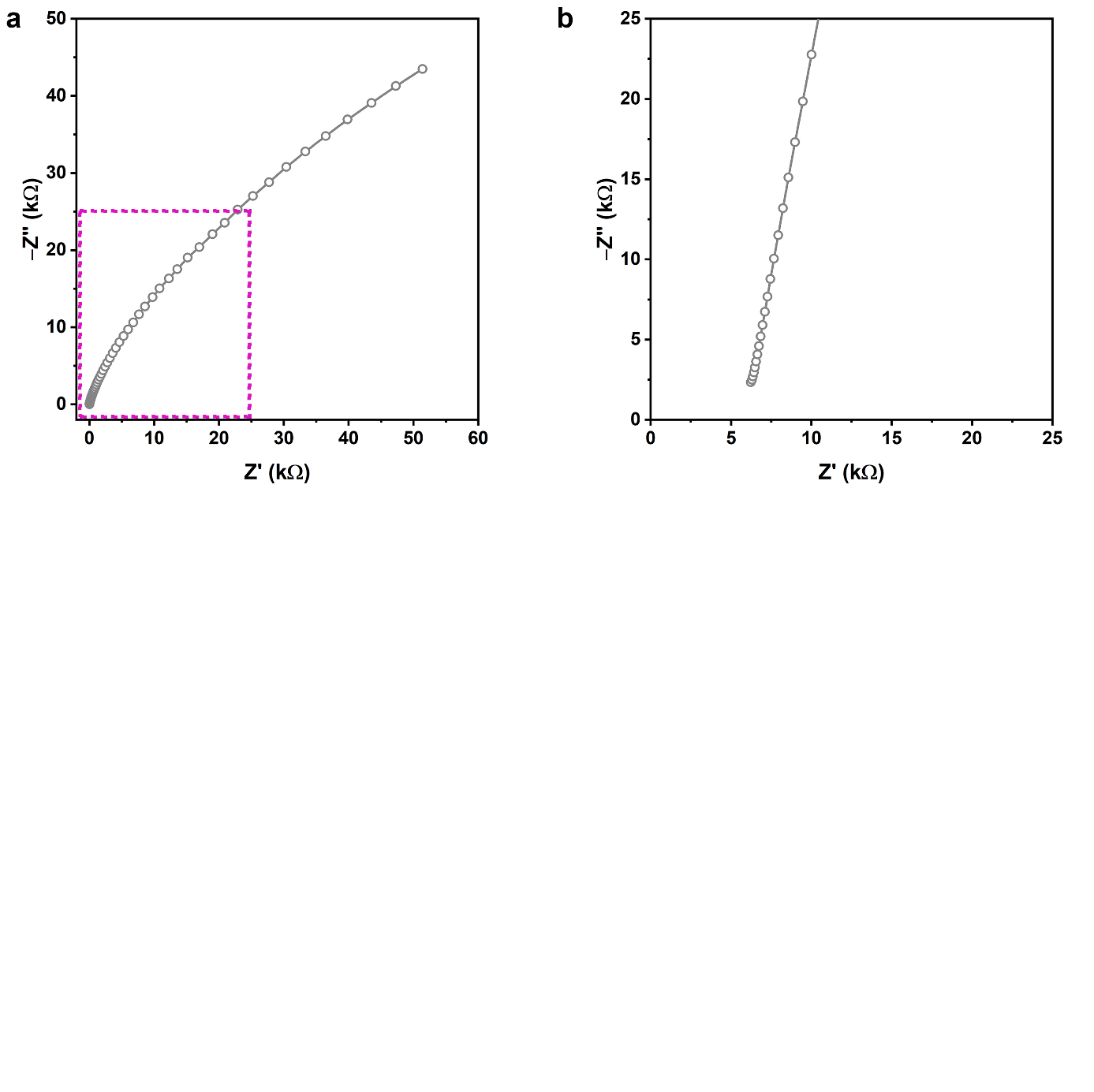


**Figure S26.** Electrochemical impedance spectroscopy (EIS) curves of solution resistance. a) Full range, b) Magnification of purple square area.


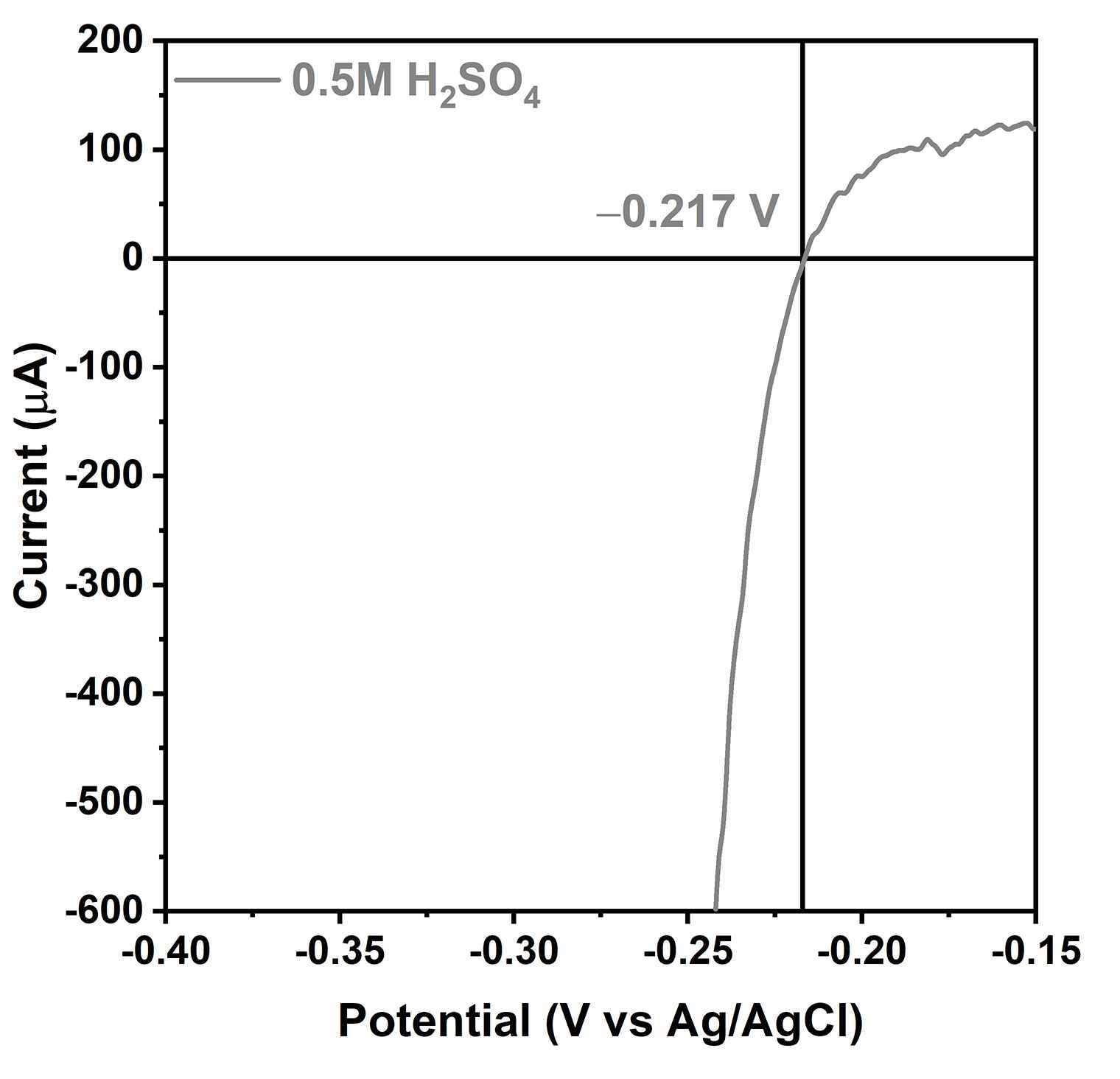


**Figure S27.** Calibration of reference electrode. Current-potential curves of Pt wire in highly pure H_2_-saturated 0.5 ᴍ aq. H_2_SO_4_ solution, used for calibrating the Ag/AgCl electrode with respect to RHE. Scan rate: 1 mV s^−1^.


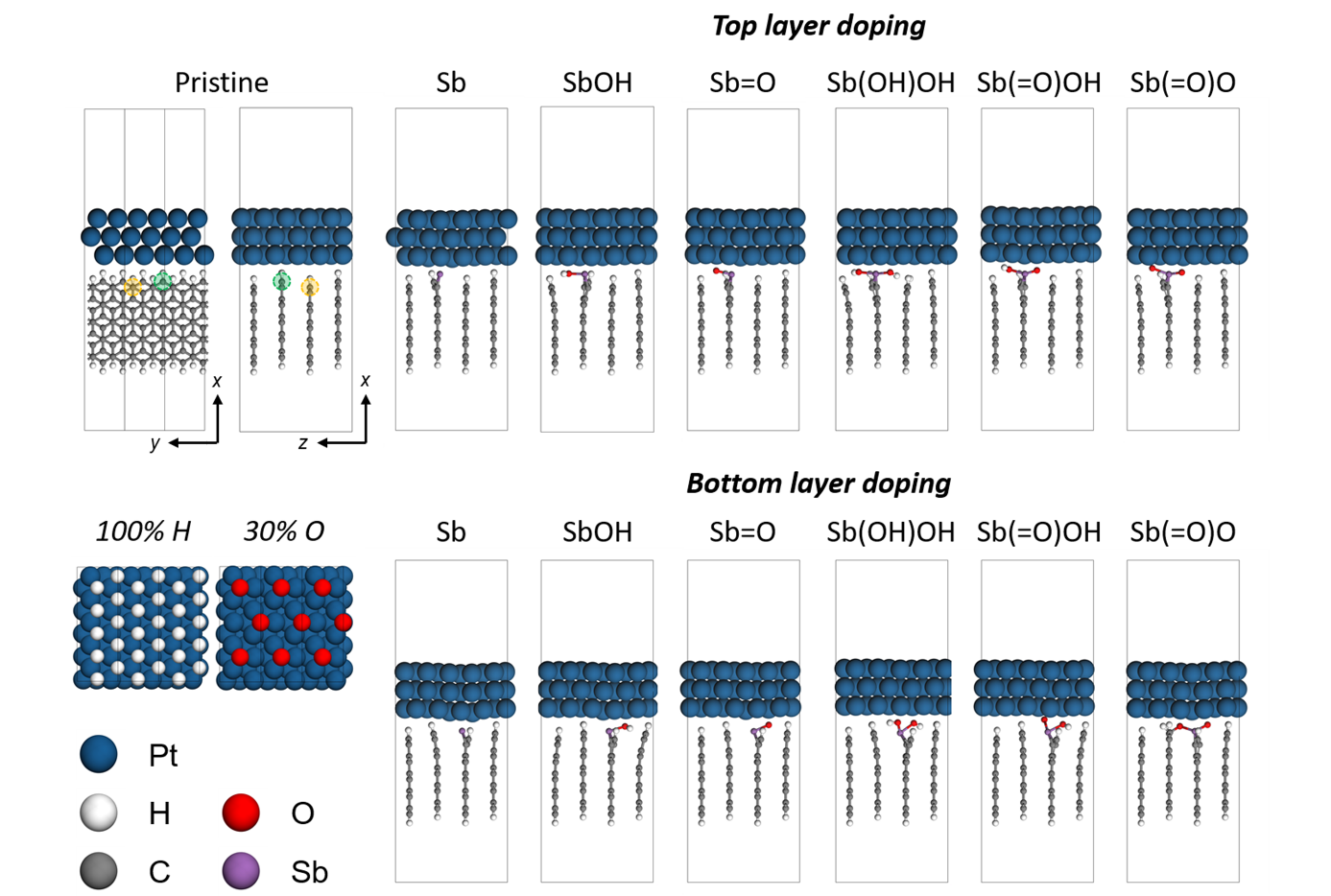


**Figure S28.** DFT-optimized structures of the PtSbGnP models. Translucent green and orange circles in the pristine model represent doping sites for Sb or oxidized Sb in the ‘Top’ and ‘Bottom’ layers, respectively. The reduced and oxidized surface conditions were considered on the top of the Pt slab. Platinum, hydrogen, carbon, oxygen, and antimony atoms are colored dark blue, white, grey, red, and purple, respectively.


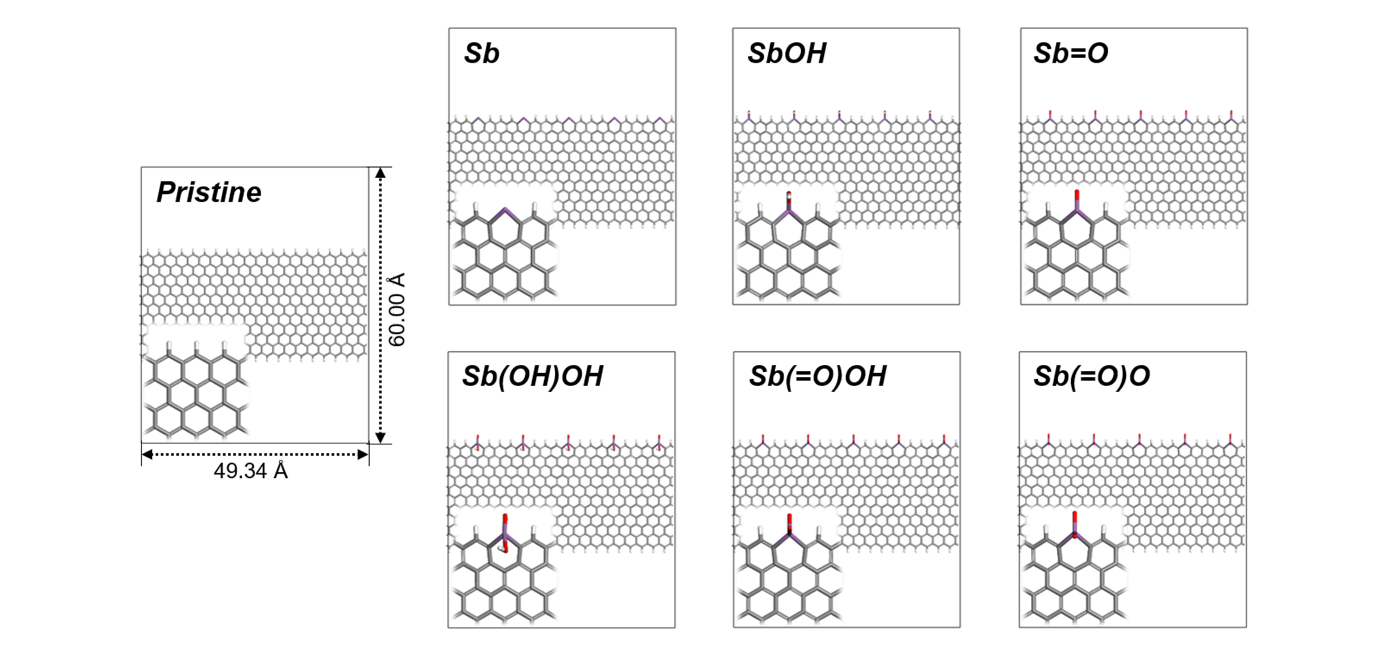


**Figure S29.** Model systems for Monte Carlo (MC) simulation. Hydrogen, carbon, oxygen, and antimony atoms are colored white, grey, red, and purple, respectively.

**Table S1.** Elemental composition of the Pt@SbGnP and SbGnP from elemental analysis and XPS

| Sample | TGA (Char yield)  in air at 1000 °C (wt%) | Element | EA  (wt%) | XPS  (at%) |
| --- | --- | --- | --- | --- |
| SbGnP | 11 | C (%) | 70.34 | 87.87 |
|  |  | H (%) | 1.14 | - |
|  |  | O (%) | 14.89 | 15.95 |
|  |  | Sb (%) | (13.63)^a)^ | 1.18 |
| Pt@SbGnP | 24 (Pt + Sb)  (Pt: 13%) | C (%) |  | 78.68 |
|  |  | H (%) |  | - |
|  |  | O (%) |  | 14.98 |
|  |  | Sb (%) |  | 2.08 |
|  |  | Pt (%) |  | 4.26 |

a) Estimated value.

**Table S2.** EXAFS fitting parameters for the Pt L_3_-edge of Pt@SbGnP

| Sample | Shell | N^a^ | R  (Å)^b^ | Debye-Waller factor  (10^−3^ σ^2^ Å^−2^) | R factor |
| --- | --- | --- | --- | --- | --- |
| Pt foil | Pt-Pt | 12 | 2.76 | 0.00457 | 0.004 |
| Pt@SbGnP | Pt-O | 1.1 | 1.95 | 0.00369 | 0.013 |
|  | Pt-Sb | 2.5 | 2.54 | 0.01492 |  |
|  | Pt-Pt | 6.6 | 2.76 | 0.0052 |  |

^a^*N*: coordination numbers; ^b^*R*: bond distance.

**Table S3.** The hydrogen production rate of catalysts^a)^

| Constant  Current | 5 mA | | 10 mA | | 15 mA | |
| --- | --- | --- | --- | --- | --- | --- |
| Time (h) | Pt/C  (µmol h^−1^) | Pt@SbGnP  (µmol h^−1^) | Pt/C  (µmol h^−1^) | Pt@SbGnP  (µmol h^−1^) | Pt/C  (µmol h^−1^) | Pt@SbGnP  (µmol h^−1^) |
| 1 | 90.45  (+0.12/–0.08) | 91.95  (+0.41/–0.38) | 177.63  (+1.04/–0.64) | 179.68  (+0.38/–0.47) | 262.45  (+0.12/–0.12) | 268.86  (+0.47/–0.47) |
| 2 | 90.01  (+0.2/–0.2) | 91.66  (+0.23/–0.26) | 176.1  (+0.91/–1.39) | 180.27  (+1.44/–1.47) | 262.38  (+0.94/–1.17) | 268.55  (+0.22/–0.25) |
| 3 | 90.57  (+0.53/–0.59) | 91.53  (+0.3/–0.33) | 176.59  (+1.31/–0.88) | 180.03  (+0.23/–0.43) | 262.57  (+0.66/–0.33) | 268.97  (+0.28/–0.32) |
| 4 | 89.83  (+1.11/–1.43) | 91.78  (+0.31/–0.53) | 177.18  (+0.82/–0.67) | 180.15  (+0.62/–0.77) | 262.4  (+0.33/–0.63) | 268.74  (+0.47/–0.51) |
| 5 | 89.95  (+1.01/–0.6) | 91.94  (+0.29/–0.52) | 176.42  (+0.57/–0.4) | 179.99  (+0.36/–0.33) | 261.99  (+0.7/–0.37) | 268.79  (+0.17/–0.29) |
| 6 | 90.08  (+0.32/–0.22) | 91.59  (+0.31/–0.33) | 177.78  (+0.55/–0.6) | 180.72  (+0.27/–0.44) | 261.93  (+0.74/–0.39) | 268.45  (+0.4/–0.41) |
| 7 | 89.99  (+0.38/–0.32) | 91.35  (+0.47/–0.32) | 177.9  (+1.05/–1.92) | 180.4  (+0.74/–0.43) | 261.35  (+1.45/–1.63) | 269.01  (+0.11/–0.15) |
| 8 | 89.93  (+0.13/–0.14) | 92.13  (+0.23/–0.23) | 178.2  (+0.83/–1.15) | 180.56  (+0.37/–0.36) | 262.88  (+0.51/–0.32) | 268.99  (+0.17/–0.12) |
| 9 | 90.5  (+0.5/–0.45) | 91.74  (+0.46/–0.62) | 177.56  (+0.96/–0.86) | 180.2  (+1.03/–0.63) | 261.86  (+0.69/–0.78) | 269.18  (+0.6/–0.4) |
| 10 | 89.97  (+0.38/–0.75) | 91.66  (+0.21/–0.27) | 176.44  (+1.49/–1.26) | 180.37  (+0.3/–0.55) | 262.61  (+1.03/–0.56) | 269  (+0.35/–0.5) |
| 11 | 90.3  (+0.22/–0.19) | 91.67  (+0.13/–0.08) | 177.69  (+0.25/–0.13) | 180.09  (+0.66/–0.36) | 261.72  (+0.19/–0.37) | 269.2  (+0.36/–0.6) |
| 12 | 89.98  (+0.55/–0.42) | 91.73  (+0.54/–0.42) | 177.09  (+0.62/–0.7) | 180.59  (+0.55/–0.6) | 261.86  (+0.55/–0.72) | 269.3  (+1.04/–0.87) |
| 13 | 90.24  (+0.23/–0.31) | 91.38  (+0.16/–0.31) | 176.36  (+1.53/–1.28) | 179.61  (+0.48/–0.69) | 261.17  (+0.28/–0.49) | 269  (+0.33/–0.37) |
| 14 | 90.19  (+0.34/–0.34) | 91.6  (+0.43/–0.54) | 177.64  (+0.65/–0.39) | 180.56  (+0.64/–1.07) | 261.09  (+0.21/–0.4) | 268.7  (+0.52/–0.53) |
| 15 | 90.3  (+0.72/–0.5) | 91.81  (+0.21/–0.3) | 177.32  (+1.28/–0.91) | 180.38  (+0.26/–0.5) | 261.81  (+1.59/–1.53) | 268.64  (+0.25/–0.34) |
| 16 | 90.1  (+0.15/–0.14) | 91.54  (+0.26/–0.29) | 177.93  (+0.29/–0.17) | 180.27  (+0.22/–0.35) | 263.01  (+1.07/–0.76) | 268.99  (+0.14/–0.19) |
| 17 | 89.36  (+0.32/–0.51) | 91.63  (+0.56/–0.35) | 177.31  (+0.8/–0.84) | 180.43  (+0.12/–0.19) | 261.07  (+0.93/–1.53) | 268.63  (+0.18/–0.35) |
| 18 | 90.11  (+0.52/–0.53) | 91.32  (+0.38/–0.34) | 177.11  (+1.51/–1.69) | 180.38  (+0.46/–0.59) | 261.75  (+0.53/–0.69) | 268.49  (+0.3/–0.58) |
| 19 | 90.19  (+0.29/–0.3) | 91.8  (+0.29/–0.2) | 178.43  (+1.41/–0.89) | 179.93  (+0.3/–0.47) | 262.37  (+1.51/–1.07) | 268.92  (+0.21/–0.16) |
| 20 | 90.6  (+0.15/–0.29) | 91.87  (+0.1/–0.14) | 176.76  (+1.78/–1) | 180.01  (+0.81/–1.1) | 261.8  (+0.87/–1.24) | 268.86  (+0.43/–0.56) |

^a)^ This value is an average of three electrode test results.

**Table S4.** Amount of hydrogen production of catalysts^a)^

| Constant  Current | 5 mA | | 10 mA | | 15 mA | |
| --- | --- | --- | --- | --- | --- | --- |
| Time (h) | **Pt/C**  **(µmol)** | **Pt@SbGnP**  **(µmol)** | **Pt/C**  **(µmol)** | **Pt@SbGnP**  **(µmol)** | **Pt/C**  **(µmol)** | **Pt@SbGnP**  **(µmol)** |
| 1 | 90.45  (+0.12/–0.08) | 91.95  (+0.41/–0.38) | 177.63  (+1.04/–0.64) | 179.68  (+0.38/–0.47) | 262.45  (+0.12/–0.12) | 268.86  (+0.47/–0.47) |
| 2 | 180.46  (+0.32/–0.24) | 183.61  (+0.14/–0.16) | 353.73  (+1.52/–1.79) | 359.96  (+1.82/–1.94) | 524.83  (+0.94/–1.05) | 537.41  (+0.69/–0.72) |
| 3 | 271.03  (+0.45/–0.83) | 275.13  (+0.17/–0.32) | 530.31  (+1.08/–0.6) | 539.99  (+2.05/–2.37) | 787.4  (+1.6/–1.38) | 806.38  (+0.96/–1.04) |
| 4 | 360.86  (+1.56/–1.05) | 366.92  (+0.46/–0.36) | 707.49  (+0.94/–1.27) | 720.14  (+2.67/–2.22) | 1049.79  (+1.94/–2.01) | 1075.12  (+1/–1.55) |
| 5 | 450.81  (+1.15/–1.11) | 458.86  (+0.75/–0.88) | 883.91  (+0.54/–0.7) | 900.13  (+3.03/–2.25) | 1311.78  (+1.56/–1.32) | 1343.91  (+1.11/–1.83) |
| 6 | 540.9  (+1.47/–1.33) | 550.45  (+1.06/–0.86) | 1061.7  (+1.09/–0.66) | 1080.84  (+3.3/–2.07) | 1573.71  (+2.3/–1.71) | 1612.36  (+1.51/–2.24) |
| 7 | 630.88  (+1.85/–1.65) | 641.8  (+1.53/–1.19) | 1239.59  (+1.96/–2.36) | 1261.25  (+4.03/–2.38) | 1835.06  (+0.86/–1.53) | 1881.37  (+1.54/–2.39) |
| 8 | 720.82  (+1.86/–1.52) | 733.94  (+1.53/–1.42) | 1417.8  (+2.28/–3.51) | 1441.81  (+4.02/–2.74) | 2097.94  (+0.67/–1.02) | 2150.37  (+1.43/–2.45) |
| 9 | 811.32  (+1.41/–1.01) | 825.68  (+0.91/–1.26) | 1595.35  (+2.19/–3.61) | 1622.01  (+5.05/–3.36) | 2359.8  (+1.03/–1.8) | 2419.55  (+2.03/–2.85) |
| 10 | 901.29  (+0.66/–0.63) | 917.34  (+1.12/–1.2) | 1771.79  (+1.96/–2.11) | 1802.39  (+5.35/–3.92) | 2622.4  (+1.8/–2.27) | 2688.55  (+1.53/–2.7) |
| 11 | 991.58  (+0.88/–0.67) | 1009  (+1.04/–1.25) | 1949.48  (+2.21/–2.24) | 1982.48  (+6.01/–4.28) | 2884.12  (+1.98/–2.64) | 2957.75  (+1.53/–2.46) |
| 12 | 1081.56  (+0.46/–0.34) | 1100.73  (+1.58/–1.37) | 2126.58  (+2.84/–2.17) | 2163.07  (+6.05/–4.88) | 3145.98  (+2.53/–3.36) | 3227.05  (+2.57/–2.63) |
| 13 | 1171.8  (+0.54/–0.43) | 1192.11  (+1.75/–1.68) | 2302.93  (+2.59/–3.45) | 2342.68  (+6.53/–4.66) | 3407.15  (+2.82/–3.86) | 3496.05  (+2.61/–2.3) |
| 14 | 1261.99  (+0.2/–0.11) | 1283.71  (+1.86/–2.22) | 2480.57  (+2.2/–2.8) | 2523.24  (+7.18/–4.24) | 3668.24  (+2.42/–3.65) | 3764.74  (+3.13/–2.29) |
| 15 | 1352.29  (+0.61/–0.31) | 1375.52  (+1.95/–2.01) | 2657.89  (+1.83/–1.51) | 2703.62  (+7.42/–4.74) | 3930.05  (+4.01/–5.18) | 4033.39  (+3.37/–2.19) |
| 16 | 1442.39  (+0.47/–0.32) | 1467.06  (+1.98/–1.75) | 2835.82  (+2.12/–1.63) | 2883.89  (+7.56/–4.52) | 4193.06  (+5.09/–5.49) | 4302.37  (+3.42/–2.05) |
| 17 | 1531.75  (+0.66/–0.66) | 1558.69  (+1.77/–2.1) | 3013.13  (+1.27/–1.59) | 3064.33  (+7.68/–4.71) | 4454.13  (+5.69/–7.03) | 4571  (+3.6/–2.4) |
| 18 | 1621.86  (+0.52/–0.66) | 1650.01  (+2.15/–2.14) | 3190.24  (+0.49/–0.42) | 3244.71  (+8.14/–4.58) | 4715.88  (+6.22/–7.71) | 4839.49  (+3.9/–2.98) |
| 19 | 1712.04  (+0.21/–0.36) | 1741.82  (+1.96/–2.24) | 3368.67  (+1.91/–1.31) | 3424.64  (+8.31/–4.28) | 4978.26  (+7.74/–8.78) | 5108.41  (+3.84/–2.77) |
| 20 | 1802.64  (+0.36/–0.22) | 1833.69  (+2.06/–2.21) | 3545.44  (+1.19/–2.09) | 3604.65  (+9.12/–5.13) | 5240.06  (+8.1/–10.03) | 5377.27  (+3.28/–2.34) |

^a)^ This value is an average of three electrode test results.

**Table S5.** Faradaic efficiency of catalysts^a)^

| **Constant**  **Current** | **5 mA** | | **10 mA** | | **15 mA** | |
| --- | --- | --- | --- | --- | --- | --- |
| **Time (h)** | **Pt/C**  **(%)** | **Pt@SbGnP**  **(%)** | **Pt/C**  **(%)** | **Pt@SbGnP**  **(%)** | **Pt/C**  **(%)** | **Pt@SbGnP**  **(%)** |
| **1** | 96.97  (+0.13/–0.09) | 98.57  (+0.44/–0.41) | 95.22  (+0.56/–0.34) | 96.31  (+0.2/–0.25) | 93.79  (+0.04/–0.04) | 96.08  (+0.17/–0.17) |
| **2** | 96.49  (+0.21/–0.21) | 98.27  (+0.24/–0.28) | 94.39  (+0.49/–0.74) | 96.63  (+0.77/–0.79) | 93.76  (+0.34/–0.42) | 95.97  (+0.08/–0.09) |
| **3** | 97.1  (+0.57/–0.63) | 98.12  (+0.33/–0.35) | 94.66  (+0.7/–0.47) | 96.5  (+0.13/–0.23) | 93.83  (+0.24/–0.12) | 96.12  (+0.1/–0.11) |
| **4** | 96.3  (+1.19/–1.53) | 98.4  (+0.34/–0.57) | 94.97  (+0.44/–0.36) | 96.57  (+0.33/–0.41) | 93.77  (+0.12/–0.22) | 96.04  (+0.17/–0.18) |
| **5** | 96.43  (+1.09/–0.64) | 98.57  (+0.31/–0.56) | 94.57  (+0.31/–0.21) | 96.48  (+0.19/–0.18) | 93.62  (+0.25/–0.13) | 96.05  (+0.06/–0.1) |
| **6** | 96.58  (+0.35/–0.23) | 98.19  (+0.33/–0.35) | 95.3  (+0.3/–0.32) | 96.87  (+0.14/–0.24) | 93.6  (+0.26/–0.14) | 95.93  (+0.14/–0.15) |
| **7** | 96.47  (+0.41/–0.34) | 97.93  (+0.51/–0.35) | 95.36  (+0.56/–1.03) | 96.7  (+0.39/–0.23) | 93.39  (+0.52/–0.58) | 96.13  (+0.04/–0.05) |
| **8** | 96.42  (+0.14/–0.15) | 98.77  (+0.25/–0.25) | 95.52  (+0.45/–0.62) | 96.79  (+0.2/–0.19) | 93.94  (+0.18/–0.12) | 96.13  (+0.06/–0.04) |
| **9** | 97.02  (+0.54/–0.48) | 98.35  (+0.5/–0.67) | 95.18  (+0.51/–0.46) | 96.59  (+0.55/–0.34) | 93.58  (+0.24/–0.28) | 96.19  (+0.22/–0.14) |
| **10** | 96.45  (+0.41/–0.81) | 98.27  (+0.23/–0.29) | 94.58  (+0.8/–0.68) | 96.68  (+0.16/–0.3) | 93.84  (+0.37/–0.2) | 96.13  (+0.13/–0.18) |
| **11** | 96.8  (+0.24/–0.2) | 98.27  (+0.13/–0.09) | 95.25  (+0.13/–0.07) | 96.53  (+0.35/–0.19) | 93.52  (+0.07/–0.13) | 96.2  (+0.13/–0.22) |
| **12** | 96.46  (+0.59/–0.45) | 98.34  (+0.58/–0.45) | 94.93  (+0.33/–0.37) | 96.8  (+0.3/–0.32) | 93.58  (+0.2/–0.26) | 96.24  (+0.37/–0.31) |
| **13** | 96.74  (+0.25/–0.34) | 97.96  (+0.18/–0.33) | 94.53  (+0.82/–0.69) | 96.27  (+0.26/–0.37) | 93.33  (+0.1/–0.18) | 96.13  (+0.12/–0.13) |
| **14** | 96.69  (+0.37/–0.37) | 98.2  (+0.46/–0.58) | 95.22  (+0.35/–0.21) | 96.79  (+0.35/–0.57) | 93.3  (+0.07/–0.14) | 96.02  (+0.19/–0.19) |
| **15** | 96.8  (+0.77/–0.54) | 98.43  (+0.22/–0.32) | 95.05  (+0.69/–0.49) | 96.69  (+0.14/–0.27) | 93.56  (+0.57/–0.55) | 96  (+0.09/–0.12) |
| **16** | 96.6  (+0.16/–0.15) | 98.14  (+0.28/–0.31) | 95.37  (+0.16/–0.09) | 96.63  (+0.12/–0.19) | 93.99  (+0.38/–0.27) | 96.12  (+0.05/–0.07) |
| **17** | 95.79  (+0.34/–0.55) | 98.24  (+0.6/–0.38) | 95.04  (+0.43/–0.45) | 96.72  (+0.06/–0.1) | 93.3  (+0.33/–0.55) | 95.99  (+0.06/–0.13) |
| **18** | 96.6  (+0.56/–0.56) | 97.9  (+0.41/–0.36) | 94.94  (+0.81/–0.91) | 96.69  (+0.25/–0.32) | 93.54  (+0.19/–0.25) | 95.95  (+0.11/–0.21) |
| **19** | 96.69  (+0.31/–0.32) | 98.42  (+0.31/–0.21) | 95.65  (+0.76/–0.48) | 96.45  (+0.16/–0.25) | 93.76  (+0.54/–0.38) | 96.1  (+0.08/–0.06) |
| **20** | 97.13  (+0.16/–0.31) | 98.49  (+0.11/–0.14) | 94.75  (+0.96/–0.53) | 96.49  (+0.43/–0.59) | 93.55  (+0.31/–0.44) | 96.08  (+0.15/–0.2) |

^a)^ This value is an average of three electrode test results.

**Table S6.** Average value of evaluation parameters of catalysts^a)^

| Constant  Current | 5 mA | | 10 mA | | 15 mA | |
| --- | --- | --- | --- | --- | --- | --- |
| Parameter | **Pt/C** | **Pt@SbGnP** | **Pt/C** | **Pt@SbGnP** | **Pt/C** | **Pt@SbGnP** |
| Hydrogen Production rate  (µmol h^−1^) | 90.13  (+0.02/–0.01) | 91.68  (+0.1/–0.11) | 177.27  (+0.06/–0.1) | 180.23  (+0.46/–0.26) | 262  (+0.41/–0.5) | 268.86  (+0.16/–0.12) |
| Amount of hydrogen production  (µmol) | **@ 10 hours**  901.29  (+0.66/–0.63)  **@ 20 hours**  1802.64  (+0.36/–0.22) | **@ 10 hours**  917.34  (+1.12/–1.2)  **@ 20 hours**  1833.69  (+2.06/–2.21) | **@ 10 hours**  1771.79  (+1.96/–2.11)  **@ 20 hours**  3545.44  (+1.19/–2.09) | **@ 10 hours**  1802.39  (+5.35/–3.92)  **@ 20 hours**  3604.65  (+9.12/–5.13) | **@ 10 hours**  2622.4  (+1.8/–2.27)  **@ 20 hours**  5240.06  (+8.1/–10.03) | **@ 10 hours**  2688.55  (+1.53/–2.7)  **@ 20 hours**  5377.27  (+3.28/–2.34) |
| Faradaic efficiency  (%) | 96.63  (+0.02/–0.01) | 98.29  (+0.11/–0.12) | 95.02  (+0.03/–0.06) | 96.61  (+0.24/–0.14) | 93.63  (+0.14/–0.18) | 96.08  (+0.06/–0.04) |
| Hydrogen production per power consumption  (L kWh^−1^) | 256.77  (+0.05/–0.03) | 263.99  (+0.3/–0.32) | 244.14  (+0.08/–0.14) | 251.95  (+0.64/–0.36) | 234.48  (+0.36/–0.45) | 244.95  (+0.15/–0.11) |
| Hydrogen production cost^b)^  (USD kg^−1^) | 3.1 | 3.0 | 3.3 | 3.2 | 3.4 | 3.3 |

^a)^ This value is an average of three electrode test results.

^b)^ Hydrogen (1L = 0.0892g) and 0.071 USD kWh^−1^ (business electricity price of South Korea)

**Table S7.** Comparison of single cell performance of this work and reported works

| Cathode material | Anode material | Cathode loading (mg cm^−2^) | Anode loading (mg cm^−2^) | V@1A (V) | V@1A per cathode loading  (V cm^2^ mg_pt_^−1^) | Reference |
| --- | --- | --- | --- | --- | --- | --- |
| Pt@SbGnP | **IrO_2_** | 1 | 1 | 1.74 | 13.38 | This work |
| Pt/C | **IrO_2_** | 1 | 1 | 1.76 | 8.8 | This work |
| 40% Pt/GNF | **Ir-Black** | 0.8 | 2 | 1.67 | 5.22 | Int. J. Hydrogen Energy 36 (2011) 4143–4147 |
| 40% Pt/XC-72 | **Ir-Black** | 0.8 | 2 | 1.7 | 5.31 | Int. J. Hydrogen Energy 36 (2011) 4143–4147 |
| Pt40/Vulcan XC-72 | **Ir-Black** | 0.7 | 2.4 | 1.66 | 5.93 | J. Power Sources 177 (2008) 281–285 |
| Pd40/Vulcan XC-72 | **Ir-Black** | 0.7 | 2.4 | 1.7 | 6.07 | J. Power Sources 177 (2008) 281–285 |
| Pt-black | **Ir-Black** | 0.8 | 2 | 1.71 | 2.14 | Int. J. Hydrogen Energy 34 (2009) 5986–5991 |
| Pt-black | **IrO_2_** | 2.5 | 2 | 1.6 | 0.64 | Electrochem. Com. 9 (2007) 667–670 |
| 40% Pt/C | **RuO_2_** | 0.4 | 10 | 1.88 | 11.75 | J. Mol. Catal. A:  Chem. 247 (2006) 7–13 |
| 30% Pt/C | **RuO_2_** | 0.5 | 3 | 1.65 | 11 | Int. J. Hydrogen Energy 33 (2008) 4955–4961 |
| 30% Pt/C | **RuO_2_** | 0.5 | 1.5 | 1.63 | 10.87 | Electrochim.  Acta 54 (2009) 6250–6256 |
| 30% Pt/C | **IrO_2_** | 0.5 | 1.5 | 1.67 | 11.13 | Electrochim.  Acta 54 (2009) 6250–6256 |
| 60% Pt/C | **IrO_2_** | 0.5 | 3 | 1.58 | 5.27 | Trans. Faraday Soc. 67 (1971) 3550–3557 |
| 30% Pt/C | **IrO_2_** | 0.5 | 2.5 | 1.7 | 11.33 | Electrochem. Commun. 13 (2011) 437–439 |
| 40% Pt/C | **Ru_0.7_Ir_0.3_O_2_** | 0.5 | 2.5 | 1.7 | 8.5 | Int. J. Hydrogen Energy 32 (2007) 2320–2324 |
| 40% Pt/C | **IrO_2_/SnO_2_** | 0.5 | 1.5 | 1.57 | 7.85 | Int. J. Energy Res. 37 (2013) 875–883. |
| 40% Pt/C | **RuO_2_/SnO_2_** | 0.6 | 3 | 1.723 | 7.18 | Int. J. Energy Res. 37 (2013) 875–883. |
| 40% Pt/C | **RuO_2_** | 0.6 | 3 | 1.74 | 7.25 | Int. J. Energy Res. 37 (2013) 875–883. |
| 30% Pd/N-CNT | **RuO_2_** | 0.7 | 3 | 1.84 | 8.76 | Int. J. Hydrogen Energy 41  (2016) 20447–20454 |
| 30% Pd/P-CNT | **RuO_2_** | 0.7 | 3 | 2 | 9.52 | Int. J. Ionics 24  (2018) 3113–3121 |
| 30% Pd/PG | **RuO_2_** | 0.7 | 3 | 1.95 | 9.29 | Int. J. Green  Energy 15 (2018) 558–567 |
| 30% Pd/PN-CNPs | **RuO_2_** | 0.7 | 3 | 1.9 | 9.05 | Ionics 25 (2019) 2615-2625 |
| 30% Pt/CB | **Ru_0.8_Pd_0.2_O_2_** | 0.7 | 3 | 2.03 | 9.67 | Ionics 24 (2018) 2411–2419 |
| 20% Pt/C | **Ir_0.6_Ru_0.4_O_2_** | 2.04 | 2.04 | 1.56 | 3.82 | Int. J. Hydrogen Energy 32 (2007) 2320–2324. |
